# SHP2 blockade enhances anti-tumor immunity via tumor cell intrinsic and extrinsic mechanisms

**DOI:** 10.1038/s41598-021-80999-x

**Published:** 2021-01-14

**Authors:** Ye Wang, Morvarid Mohseni, Angelo Grauel, Javier Estrada Diez, Wei Guan, Simon Liang, Jiyoung Elizabeth Choi, Minying Pu, Dongshu Chen, Tyler Laszewski, Stephanie Schwartz, Jane Gu, Leandra Mansur, Tyler Burks, Lauren Brodeur, Roberto Velazquez, Steve Kovats, Bhavesh Pant, Giri Buruzula, Emily Deng, Julie T. Chen, Farid Sari-Sarraf, Christina Dornelas, Malini Varadarajan, Haiyan Yu, Chen Liu, Joanne Lim, Huai-Xiang Hao, Xiaomo Jiang, Anthony Malamas, Matthew J. LaMarche, Felipe Correa Geyer, Margaret McLaughlin, Carlotta Costa, Joel Wagner, David Ruddy, Pushpa Jayaraman, Nathaniel D. Kirkpatrick, Pu Zhang, Oleg Iartchouk, Kimberly Aardalen, Viviana Cremasco, Glenn Dranoff, Jeffrey A. Engelman, Serena Silver, Hongyun Wang, William D. Hastings, Silvia Goldoni

**Affiliations:** 1 Oncology Disease Area, Novartis Institutes for BioMedical Research, 250 Massachusetts Avenue, Cambridge, MA 02139 USA; 2 Exploratory Immuno-Oncology, Novartis Institutes for BioMedical Research, 250 Massachusetts Avenue, Cambridge, MA 02139 USA; 3 Chemical Biology & Therapeutics, Novartis Institutes for BioMedical Research, Cambridge, USA; 4 Analytical Sciences & Imaging, Novartis Institutes for BioMedical Research, Cambridge, USA; 5 Global Discovery Chemistry, Novartis Institutes for BioMedical Research, Cambridge, USA; 6 Oncology Disease Area, Novartis Institutes for BioMedical Research, Basel, Switzerland

**Keywords:** Cancer microenvironment, Cancer models, Cancer therapy, Tumour immunology, Cancer, Immunology, Immunotherapy, Tumour immunology

## Abstract

SHP2 is a ubiquitous tyrosine phosphatase involved in regulating both tumor and immune cell signaling. In this study, we discovered a novel immune modulatory function of SHP2. Targeting this protein with allosteric SHP2 inhibitors promoted anti-tumor immunity, including enhancing T cell cytotoxic function and immune-mediated tumor regression. Knockout of SHP2 using CRISPR/Cas9 gene editing showed that targeting SHP2 in cancer cells contributes to this immune response. Inhibition of SHP2 activity augmented tumor intrinsic IFNγ signaling resulting in enhanced chemoattractant cytokine release and cytotoxic T cell recruitment, as well as increased expression of MHC Class I and PD-L1 on the cancer cell surface. Furthermore, SHP2 inhibition diminished the differentiation and inhibitory function of immune suppressive myeloid cells in the tumor microenvironment. SHP2 inhibition enhanced responses to anti-PD-1 blockade in syngeneic mouse models. Overall, our study reveals novel functions of SHP2 in tumor immunity and proposes that targeting SHP2 is a promising strategy for cancer immunotherapy.

## Introduction

Immune checkpoint inhibitors and CAR-T cell therapies have emerged as highly effective approaches for treating cancer^1,2^. Yet, the majority of cancer patients do not respond to immunotherapy, creating a need for novel approaches^3^. Experimental and clinical exploration has led to the initiation of numerous clinical trials combining immune checkpoint blockade with targeted and conventional therapies such as radiation and chemotherapy^4^. Targeted therapies, including cell cycle inhibitors targeting CDK4/6^5,6^, MAP kinase signaling inhibitors^7^ and chromatin-modifying enzyme inhibitors targeting EZH2, HDAC and DNMT^8–11^, have been shown to favor anti-tumor immune responses and the effectiveness of immunotherapy in pre-clinical cancer models. Investigating the effect of targeted therapies on anti-tumor immunity is critical to rationally advance the design of combination therapies to improve patient outcomes.

The Src homology-2 domain-containing phosphatase 2 (SHP2), encoded by the gene *PTPN11*, is a ubiquitously expressed non-receptor tyrosine phosphatase which plays a regulatory role in signal transduction downstream of multiple receptor tyrosine kinases (RTKs) such as EGFR, FGFR and MET^12,13^. SHP2 promotes proliferation and survival of cancer cells by promoting GTP loading of RAS^14^, thereby activating RAS-MAPK signaling. SHP2 inhibition by the selective allosteric inhibitor SHP099 exhibits promising therapeutic potential in RTK/KRAS-driven cancers^15,16^. In addition to its oncogenic role in cancer cells, SHP2 is involved in multiple signaling pathways in immune cells. In T lymphocytes, SHP2 is recruited to the cytoplasmic tails of PD-1 and CTLA-4 and suppresses T cell activation by dephosphorylating TCRζ chains, ZAP70 and the costimulatory receptor CD28^17–20^. Beyond these molecular mechanisms, the role of SHP2 in the tumor microenvironment is largely unknown and its role in anti-tumor immunity remains to be explored. T cell-restricted ablation of SHP2 in a murine colon xenograft model increases anti-tumor immune responses by enhancing the function of CD8 cytotoxic T cells^21^. However, selective deletion of SHP2 in CD4 and CD8 T cells in a different study ultimately leads to melanoma progression and metastasis^22^. SHP2 is also downstream of CSF-1 signaling promoting macrophage proliferation and M2 polarization, suggesting another mechanism by which SHP2 inhibition could enhance anti-tumor immunity^23–25^. Given the complexity of the tumor microenvironment and the important role of SHP2 in cancer cell signaling, it is necessary to study the effect of inhibiting SHP2 activity in both tumor and immune cells to gain a better understanding of its therapeutic potential.

In the present study, we uncovered an immune modulatory role of SHP2 in the context of tumor-immune cell interactions and discovered that inhibiting SHP2 function triggers favorable changes in the tumor microenvironment and control of cancer progression. In cancer cells, SHP2 inhibition augmented interferon γ (IFNγ) signaling, which resulted in increased expression of its downstream targets, including chemoattractant cytokines and antigen presenting machinery. SHP2 inhibition also promoted T cell proliferation and function through its effect in cancer cells and immune suppressive myeloid cells. Furthermore, the combination of SHP2 inhibitors with anti-PD1 antibody resulted in significant regressions in tumor growth in syngeneic mouse models.

Our study describes the immune phenotypes associated with inhibiting SHP2 in cancer cells and the tumor microenvironment, supporting the promise of therapeutically inhibiting SHP2 activity in patients with molecularly and phenotypically diverse malignancies.

## Results

### SHP2 inhibition in cancer cells enhances immune cell-mediated tumor killing

**Figure 1:**
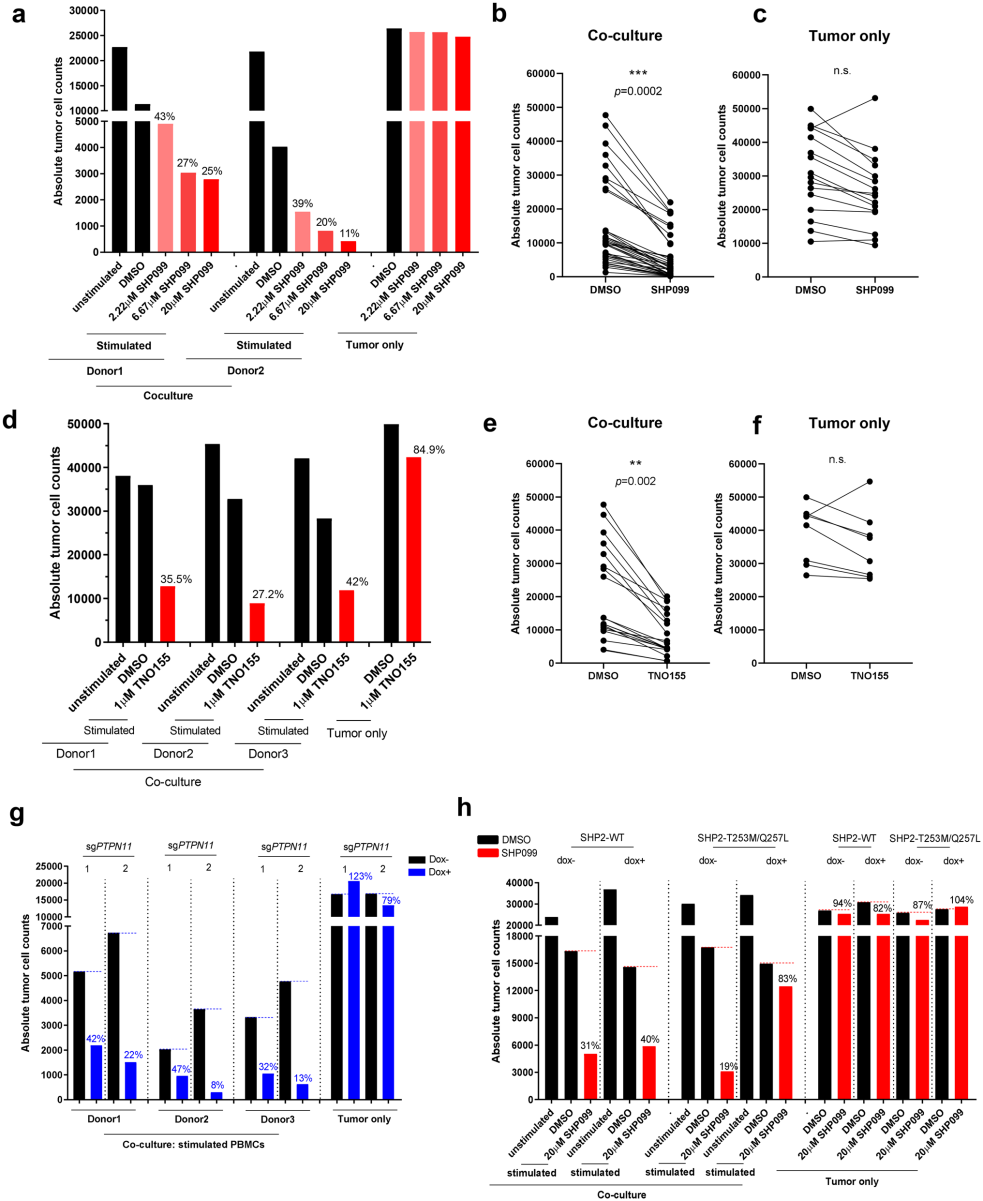
SHP2 inhibition in cancer cells enhances immune cells-mediated tumor killing. (**a**) Absolute tumor cell counts from each well of 384-well Elplasia plate after 6 days co-culture of OVCAR-8 spheroids with human PBMCs (2 donors). Relative percentage of absolute tumor counts (SHP099 treated over DMSO group) is labeled. (**b**) Absolute tumor cell counts of paired DMSO and SHP099 (20 μM) treated co-culture groups of OVCAR-8 spheroids with PBMCs from multiple replicates with different donors. (**c**) Absolute tumor cell counts of paired DMSO and SHP099 (20 μM) treated tumor only groups from multiple replicates. (**d**) Absolute tumor cell counts from each well of 384-well Elplasia plate after 6 days co-culture of OVCAR-8 spheroids with human PBMCs (3 donors). Relative percentage of absolute tumor counts (TNO155 treated over DMSO group) is labeled. (**e**) Absolute tumor cell counts of paired DMSO and TNO155 (1 μM) treated co-culture groups of OVCAR-8 spheroids with PBMCs from multiple replicates with different donors. (**f**) Absolute tumor cell counts of paired DMSO and TNO155 (1 μM) treated tumor only groups from multiple replicates. (**g**) Absolute tumor cell counts from each well of 384-well Elplasia plate after 6 days co-culture of OVCAR-8-CAS9-sg*PTPN11*-1 or OVCAR-8-CAS9-sg*PTPN11*-2 spheroids with human PBMCs (3 donors). OVCAR-8 cells were treated with or without doxycycline (100 ng/ml) for 5 days before co-culture. Relative percentage of absolute tumor counts (Dox+ over Dox− group) is labeled. (**h**) Absolute tumor cell counts from each well of 384-well Elplasia plate after 6 days co-culture of OVCAR-8-SHP2-WT or OVCAR-8-SHP2-T253M/Q257L spheroids with human PBMCs. OVCAR-8 cells were treated with or without doxycycline (100 ng/ml) for 5 days before co-culture. Relative percentage of absolute tumor counts (SHP099 treated over DMSO group) is labeled.

To explore the immune modulatory function of SHP2 in vitro, we optimized a tumor spheroid-PBMC co-culture (Supplementary Fig. 1a). The tumor spheroid format retains some physiologically relevant features of the tumor microenvironment such as oxygen and cellular proliferation gradients^26, 27^. In addition, in the context of co-cultures with activated T cells, it facilitates the study of immune cell infiltration. Initially, we tested SHP099 in the human ovarian carcinoma cell line OVCAR-8 co-cultured with freshly isolated human peripheral blood mononuclear cells (PBMCs) from healthy donors. No tumor killing was observed in co-culture in the absence of PBMC stimulation (Supplementary Fig. 1b). We activated PBMCs with anti-CD3/CD28 beads in order to simulate activation of T cells with tumor antigen. Following activation, CD3-positive T cells expanded and became the predominant immune population over 6 days (Supplementary Fig. 1c,d). When activated PBMCs were incubated with OVCAR-8 spheroids, there was substantial tumor cell killing (Fig. 1a). To assess the role of SHP2 in this process, we treated co-cultured spheroids with SHP099 and found that it decreased tumor cell numbers after 6 days, exclusively in the presence of activated T cells, in a dose-dependent manner (Fig. 1a). OVCAR-8 cells were not sensitive to SHP2 inhibition despite effective target engagement and suppression of signaling (Fig. 1a,c and Supplementary Fig. 1e). Thus, the phenotype observed in co-culture is attributed to immune cell-mediated tumor killing. The same phenotype was observed with treatment by TNO155, an allosteric SHP2 inhibitor currently in clinical development (Fig. 1d–f). Although we observed variation in baseline killing by T cells across donors, the phenotype observed following treatment with SHP2 inhibitors was reproducible (Fig. 1b,e) and consistent across a panel of cancer cell lines (Supplementary Fig. 2a–d). Ablation of antigen presenting machinery by knocking out B2M did not affect immune-mediated tumor killing (Supplementary Fig. 2e), indicating the absence of an allogeneic response.

To explore the specific contribution of inhibiting SHP2 in the cancer cells to the immune-mediated killing, we adopted a doxycycline-inducible SHP2 knockout strategy. Two single guide RNAs (sgRNAs) targeting *PTPN11* were transduced into OVCAR-8 cells constitutively expressing CAS9 protein. OVCAR-8-CAS9-sg*PTPN11*-1 and 2 cell pools showed substantial knockout efficiency (Supplementary Fig. 2f). Pools were then co-cultured with PBMCs in the presence or absence of doxycycline. Enhanced immune-mediated tumor killing was observed in doxycycline-treated co-cultures, utilizing PBMCs from 3 separate donors, indicating that SHP2 depletion in cancer cells sensitizes them to immune-mediated killing (Fig. 1g). To interrogate the effect of inhibiting SHP2 specifically in the immune cell compartment, we exogenously expressed a doxycycline-inducible SHP099-untargetable SHP2 mutant, SHP2-T253M/Q257L, in OVCAR-8 and performed co-culture with PBMCs. As cancer cells overexpressing SHP2-T253M/Q257L, but not SHP2-WT, were still able to maintain activation of MAPK signaling in the presence of SHP099, we concluded that the effect of SHP099 in this context was mostly in immune cells (Supplementary Fig. 2g). Expression of the SHP2 mutant in cancer cells dramatically attenuated immune-mediated tumor killing upon SHP099 treatment (Fig. 1h), suggesting that inhibition of SHP2 in the cancer cell is critical for SHP099 to enhance tumor cell killing by T cells. However, it should be noted that overexpression of the SHP2 mutant could not completely rescue the phenotype, suggesting that inhibition of SHP2 in immune cells may also contribute to tumor cell killing in vitro.

### SHP2 inhibition in cancer cells promotes T cell proliferation/function

**Figure 2:**
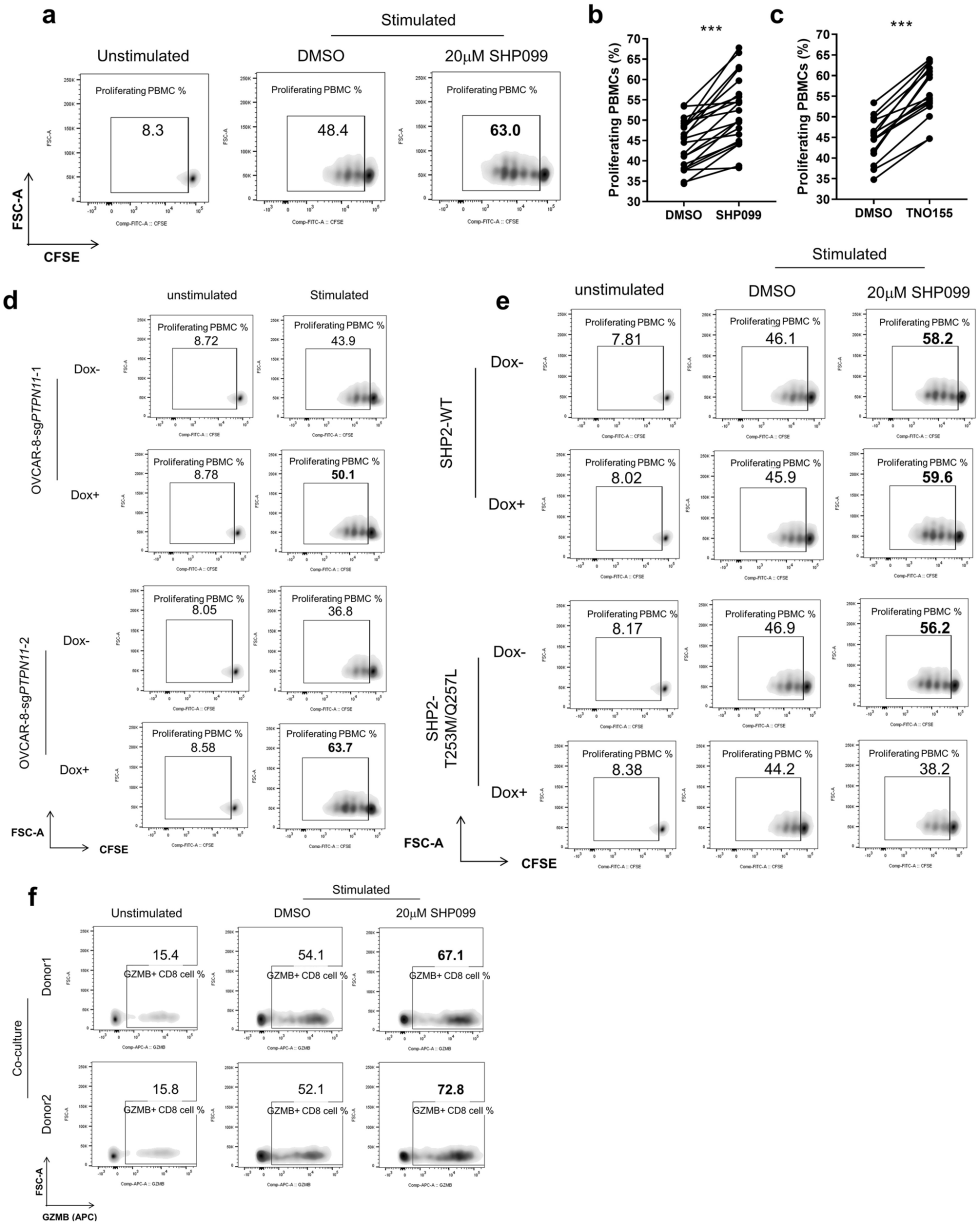
SHP2 inhibition in cancer cells promotes T cell proliferation/function. (**a**) Flow cytometry analysis of T cell proliferation in OVCAR-8-PBMC co-culture with or without SHP099 treatment. T cells with diluted CFSE signal were gated. Percentage of T cell population with diluted CFSE signal was labeled. (**b**) Percentage of proliferating T cell from paired DMSO and SHP099 (20 μM) treated co-culture groups of OVCAR-8 spheroids with PBMCs from multiple replicates with different donors. (**c**) Percentage of proliferating T cell from paired DMSO and TNO155 (1 μM) treated co-culture groups of OVCAR-8 spheroids with PBMCs from multiple replicates with different donors. (**d**) Flow cytometry analysis of T cell proliferation in co-culture of OVCAR-8-CAS9-sg*PTPN11*-1 or OVCAR-8-CAS9-sg*PTPN11*-2 spheroids with PBMCs. OVCAR-8 cells were treated with or without doxycycline (100 ng/ml) for 5 days before co-culture. T cells with diluted CFSE signal were gated. Percentage of T cell population with diluted CFSE signal was labeled. (**e**) Flow cytometry analysis of T cell proliferation in co-culture of OVCAR-8-SHP2-WT or OVCAR-8-SHP2-T253M/Q257L spheroids with PBMCs. OVCAR-8 cells were treated with or without doxycycline (100 ng/ml) for 5 days before co-culture. T cells with diluted CFSE signal were gated. Percentage of T cell population with diluted CFSE signal was labeled. (**f**) Flow cytometry analysis of intracellular staining of Granzyme B in OVCAR-8-PBMC co-culture with or without SHP099 treatment. Granzyme B-positive immune cells were gated. Percentage of Granzyme B-positive population was labeled. Flow cytometry data was analyzed and processed with FlowJo (Version 10.7.1, https://www.flowjo.com/solutions/flowjo/downloads/previous-versions).

We next assessed the effect of SHP2 inhibition on T cell proliferation in the presence or absence of OVCAR-8 cells. PBMCs were pre-labeled with carboxyfluorescein succinimidyl ester (CFSE) and analyzed by FACS to assess cell divisions. In the presence of OVCAR-8 cells, SHP2 inhibition significantly enhanced T cell proliferation in co-culture using cells from multiple donors (Fig. 2a–c). We also observed enhanced expression of Granzyme B on CD8 T cells in the SHP099 treated co-culture (Fig. 2f and Supplementary Fig. 3g). This further suggests that SHP2 inhibition enhances the tumor killing capability of cytotoxic T cells.

In the absence of tumor cells, T cell proliferation was either slightly diminished by SHP2 inhibition (in the case of SHP099) or not affected (in the case of TNO155) without affecting cell viability. (Supplementary Fig. 3a–d). It has been reported that SHP2 is a downstream mediator of immune checkpoint signaling such as PD-1 and CTLA-4, acting by dephosphorylating CD28 and ZAP70 and thus preventing TCR-mediated MAPK signaling activation^17–20^. This suggests that SHP2 inhibition might relieve the immune inhibitory effect of checkpoint signaling, activate MAPK pathway and promote T cell proliferation. However, MAPK signaling in T cells was not obviously enhanced by SHP099 treatment (Supplementary Fig. 3e), suggesting that SHP2 inhibition did not augment T cell proliferation through the MAPK pathway. We observed enhanced T cell proliferation upon SHP2 inhibition exclusively in the presence of tumor cells, indicating that the effect of SHP2 on T cells is context-dependent.

We hypothesized that inhibiting SHP2 activity in tumor cells in co-culture mediates the T cell phenotype. Indeed, doxycycline-induced SHP2 depletion in tumor cells boosted T cell proliferation in co-culture (Fig. 2d and Supplementary Fig. 3f). On the contrary, T cell proliferation was unaffected by SHP099 in co-culture with OVCAR-8 expressing SHP2 mutant (Fig. 2e).

Taken together, these data demonstrate that targeting SHP2 in tumor cells promotes T cell proliferation and killing of tumor cells, suggesting that SHP2 might have an important role in anti-tumor immunity.

### SHP2 inhibition upregulates expression of CXCR3 ligands and promotes immune infiltration in vitro

**Figure 3:**
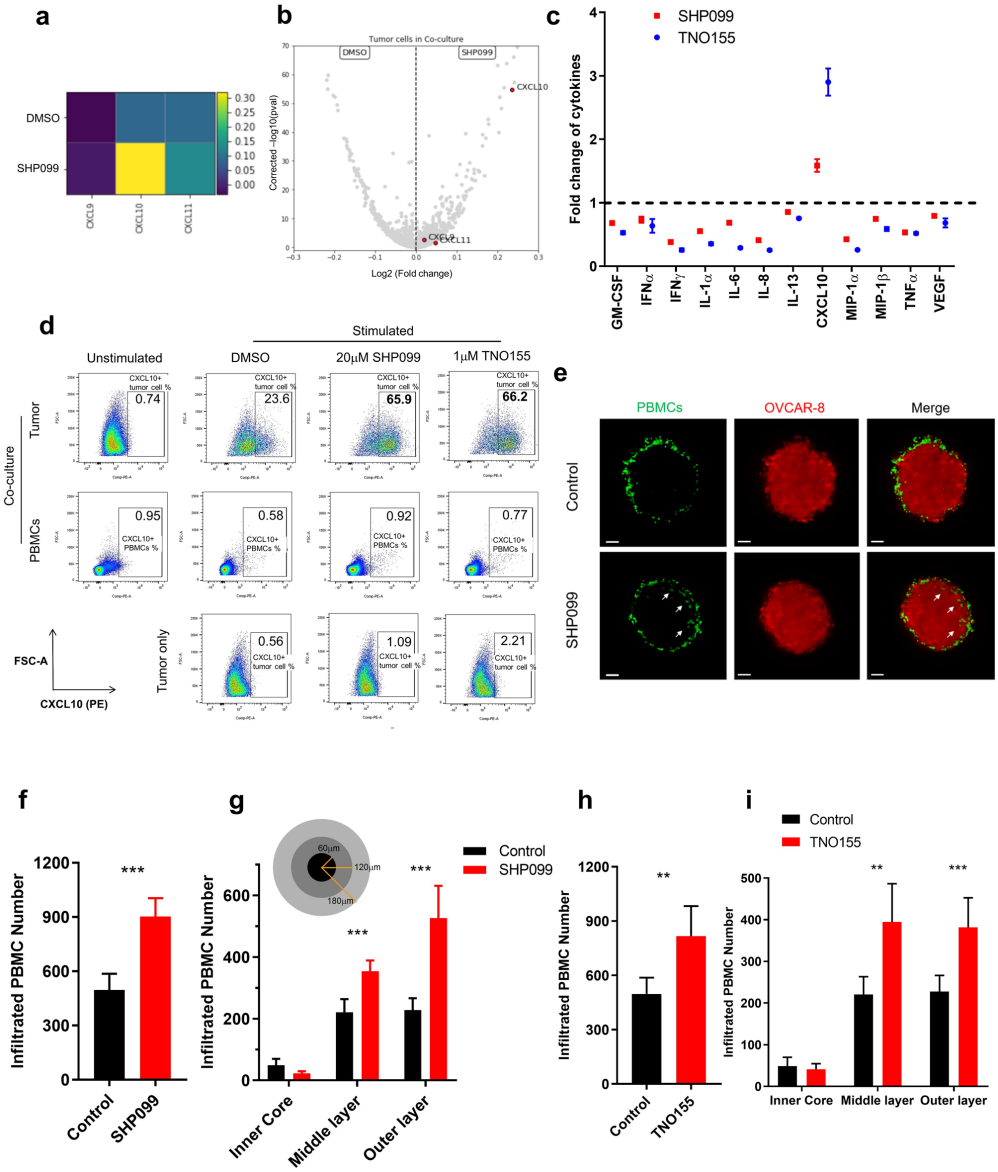
SHP2 inhibition upregulates expression of CXCR3 ligands and promotes immune cell infiltration. (**a**) Heatmap of transcriptional expression level of CXCR3 ligand genes *CXCL9*, *CXCL10*, *CXCL11* in OVCAR-8 tumor cells in co-culture from scRNAseq data. (**b**) Volcano plot of transcriptional expression level of CXCR3 ligand genes *CXCL9*, *CXCL10*, *CXCL11* in OVCAR-8 tumor cells in co-culture from scRNAseq data. (**c**) Luminex analysis of TNO155 and SHP099-induced fold change of a panel of cytokines (normalized to control group) in supernatants collected from co-culture of OVCAR-8 spheroids with PBMCs. (**d**) Flow cytometry analysis of intracellular staining of CXCL10 from 2 days of co-culture and tumor only. CXCL10-positive cells are gated. Percentage of CXCL10-positive population is labeled. (**e**) Light sheet microscopy imaging of infiltrated PBMCs (Green) inside OVCAR-8 tumor spheroids (Red) after 24 h of co-culture. Scale bar: 50 μm. (**f**, **h**) Histogram of number of total infiltrated PBMCs inside tumor spheroids. (**g**, **i**) Histogram of number of infiltrated PBMCs in different layers of tumor spheroids. Flow cytometry data was analyzed and processed with FlowJo (Version 10.7.1, https://www.flowjo.com/solutions/flowjo/downloads/previous-versions).

To define mechanisms of anti-tumor immunity elicited by SHP2 inhibition, we conducted single cell RNA sequencing (scRNAseq) of OVCAR-8 cancer cells co-cultured with PBMCs in the absence or presence of SHP099 (Supplementary Fig. 4a,b). We found that treatment with SHP099 led to increased expression of three CXCR3 ligands, the chemoattractant cytokines CXCL9, CXCL10 and CXCL11 specifically in tumor cells in co-culture, with CXCL10 showing the most significant upregulation and magnitude of expression (Fig. 3a,b). We confirmed scRNAseq data at the protein level via Luminex cytokine analysis of conditioned media from co-culture. CXCL10 secretion was enhanced by SHP099/TNO155 treatment of the co-culture (Fig. 3c). Follow-up ELISA analysis confirmed this observation (Supplementary Fig. 5a). In addition, SHP2 inhibition increased expression of CXCR3 ligands, CXCL9 and CXCL10, in MIA PaCa-2-PBMCs co-culture, suggesting that upregulation of chemoattractant cytokines by inhibition of SHP2 may be a common phenotype in the tumor microenvironment (Supplementary Fig. 5b).

To identify the cellular origin of CXCL10, we performed intracellular CXCL10 staining followed by FACS analysis. CXCL10 upregulation by SHP099/TNO155 treatment was observed exclusively in CD45-negative tumor cells in co-culture but not in immune cells, consistent with scRNAseq data (Fig. 3d).

CXCL9, CXCL10 and CXCL11 are chemoattractant cytokines for anti-tumor leukocytes that express CXCR3, such as effector T cells^28^. We explored the effect of inhibiting SHP2 on immune cell migration in vitro by imaging immune cell infiltration in tumor spheroids with light sheet fluorescence microscopy. Volume of tumor spheroids shrank over time during co-culture presumably due to immune cell-mediated killing (Supplementary Fig. 5c). Consistent with data in Fig. 1a, tumor spheroids were smaller upon SHP099 treatment compared to controls, on Day 3 and Day 6 of co-culture (Supplementary Fig. 5c,d). To avoid the confounding effect of changes in tumor spheroid size on the quantification of immune infiltration, we measured the effect of SHP099 treatment after 24 h. As most immune cells were still surrounding the surface of tumor spheroids, it was apparent that SHP099 treatment promoted infiltration of immune cells into the tumor mass (Fig. 3e, Supplementary Movie 1 and Supplementary Movie 2), with nearly twice as many infiltrated immune cells in spheroids treated with SHP099 compared to control (Fig. 3f). Further analysis dissected tumor spheroids into three regions across the radius where over 90% of infiltrated immune cells were located in the outer and middle layer of spheroids, as expected after 24 h of co-culture. SHP099 treatment augmented immune cell infiltration in both layers (Fig. 3g). The same phenotype was observed in TNO155-treated tumor spheroids (Fig. 3h,i).

### SHP2 mediates anti-tumor immunity via interferon γ signaling

From the scRNAseq data set, we compared the tumor cells from co-culture group and tumor only group based on their transcriptional profile. As Supplementary Fig. 4c showed, tumor cells from each group displayed their own clustering feature (Co-culture group tumor cells in cluster 0, 2, 4, 5, 6, 7; Tumor only group tumor cells in cluster 1, 3). Further analysis on tumor cells from co-culture revealed that SHP099-treated tumor cells specifically clustered in cluster 6 (Supplementary Fig. 4c, right). Pathway signature analysis of cluster 6 tumor cells illustrated that cytokine-mediated signaling pathway and lymphocyte proliferation/activation pathways are among the top 10 upregulated pathways, which is consistent with our results from Figs. 2 and 3 (Supplementary Fig. 4d). In addition, interferon signaling showed up multiple times as upregulated signaling in SHP099-treated tumor cells (Supplementary Fig. 4d), suggesting the involvement of interferon pathway in SHP2 inhibition-mediated immune response.

**Figure 4:**
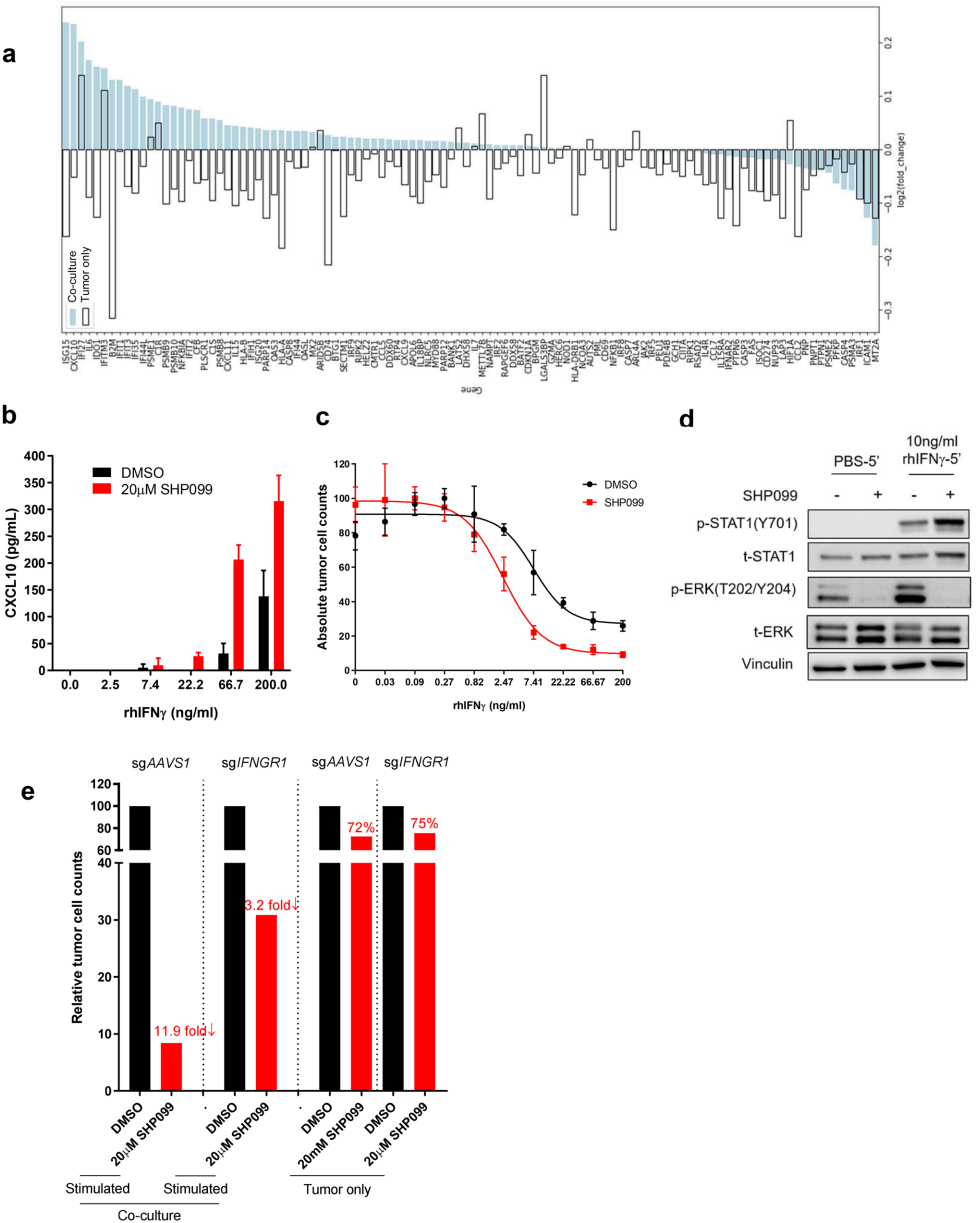
SHP2 mediates anti-tumor immunity via IFNγ signaling. (**a**) scRNAseq data showing IFNγ signaling signature genes expression level change induced by SHP099. (**b**) ELISA analysis of CXCL10 level in supernatant collected from tumor spheroids treated with IFNγ of gradient concentration in the absence or presence of SHP099 for 6 days. (**c**) Dose response curve of OVCAR-8 tumor spheroids to IFNγ treatment in the absence or presence of SHP099 after 6 days. (**d**) Immunoblotting of p-STAT1 and p-ERK in OVCAR-8 tumor spheroids after different treatments. (**e**) Absolute tumor cell counts from each well of 384-well Elplasia plate after 6 days co-culture of OVCAR-8-sg*AAVS1* or OVCAR-8-sg*IFNGR1* spheroids with PBMCs. Relative fold change of absolute tumor counts (SHP099 treated over DMSO group) is labeled.

CXCL10 and other chemoattractant cytokines are transcriptional targets of the IFNγ pathway and their expression in cancer often correlates with clinical response to immune checkpoint blockade^29–31^. We characterized gene expression changes in the IFNγ signaling pathway of tumor cells from scRNAseq data (Fig. 4a). Strikingly, a large proportion of the IFNγ signature genes were upregulated by SHP2 inhibition specifically in tumor cells in co-culture, including cytokines (CXCL9, CXCL10, CXCL11 and CCL5) and antigen presenting machinery (HLA-A, HLA-B and B2M) (Fig. 4a). Next, we quantified IFNγ concentration in co-culture conditioned media. As expected, IFNγ was detectable only upon T cell stimulation, and the overall amount of IFNγ was significantly higher in co-cultures than in cultures with only PBMCs comparably stimulated (Supplementary Fig. 6a). SHP099 treatment did not enhance IFNγ secretion from immune cells in either co-culture or PBMCs alone (Supplementary Fig. 6a,b). We hypothesized that SHP2 blockade in cancer cells could augment their response to IFNγ and tested the response of OVCAR-8 tumor spheroids to recombinant human IFNγ (rhIFNγ) in the presence of SHP099 or TNO155. Both SHP099 and TNO155 upregulated CXCL10 secretion upon rhIFNγ treatment (Fig. 4b and Supplementary Fig. 6c); in addition, SHP2 blockade lowered the dose of rhIFNγ required to affect OVCAR-8 cell viability, indicating that SHP2 inhibition sensitized tumor spheroids to IFNγ (Fig. 4c and supplementary Fig. 6d).

Downstream signaling of IFNγ is mediated through the JAK-STAT pathway^32^. Given that SHP2 negatively regulates the JAK-STAT pathway by dephosphorylating STAT1^33,34^, we performed western blots to assess STAT1 phosphorylation in OVCAR-8 cells treated with rhIFNγ in the absence or presence of SHP099. We found that treatment with SHP099 upregulated rhIFNγ-induced phosphorylation of STAT1 while blocking p-ERK activation (Fig. 4d).

Furthermore, we knocked out *IFNGR1* in OVCAR-8. Almost 75% of IFNGR1 protein was depleted in the cell pool (Supplementary Fig. 6e). In the context of co-cultures, tumor cell killing in the presence of SHP099 was considerably attenuated with *IFNGR1* knockout OVCAR-8 (Fig. 4e). This supports the hypothesis that SHP2 may enhance resistance of tumor cells to immune-mediated killing via negatively regulating IFNγ signaling, and that SHP2 blockade may function to release this inhibition.

### SHP2 inhibition enhances major histocompatibility complex (MHC) class I and programmed death-ligand 1 (PD-L1) protein expression in cancer cells through IFNγ signaling

**Figure 5:**
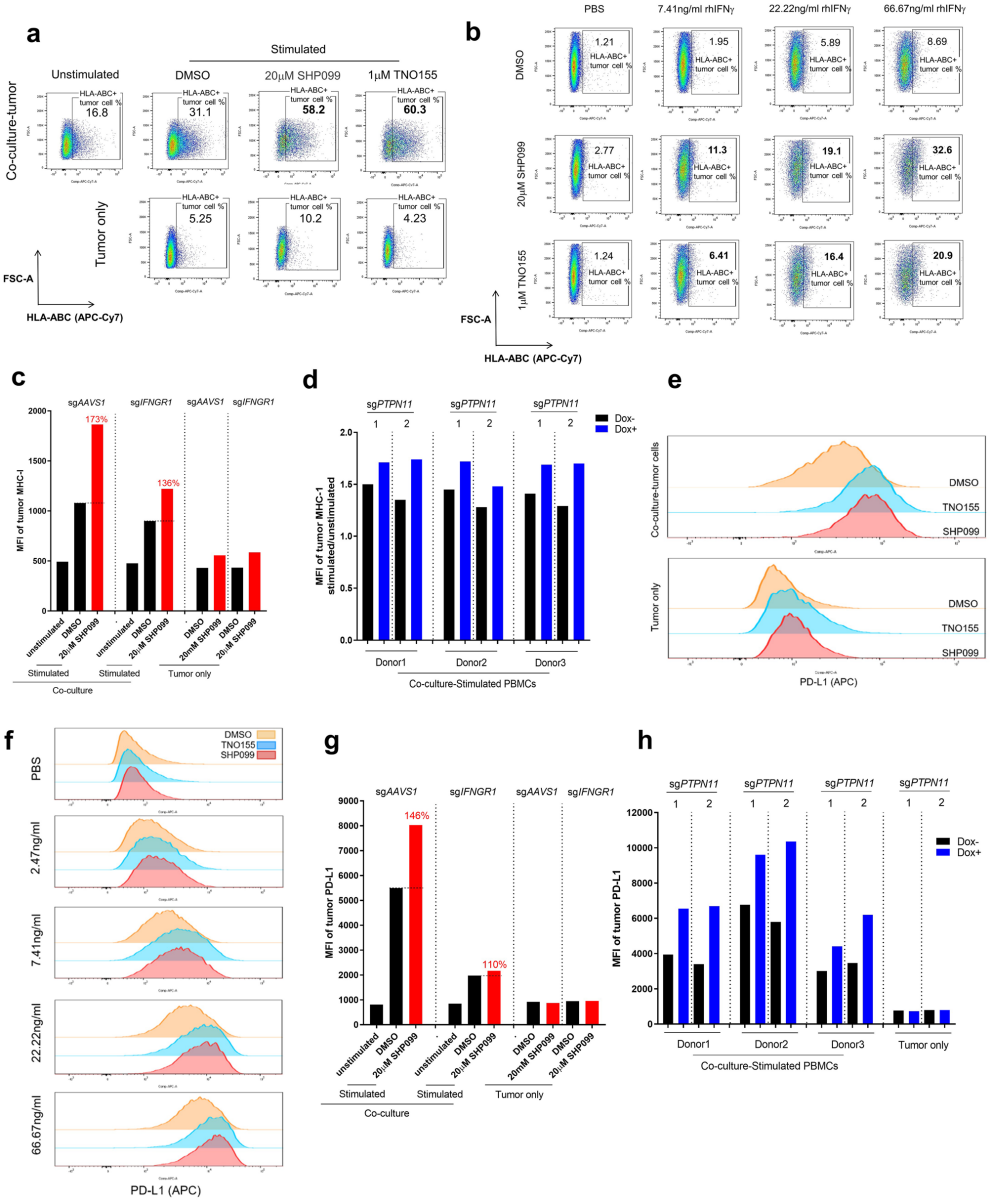
SHP2 inhibition enhances MHC class I and PD-L1 expression in cancer cells through IFNγ signaling. (**a**) Flow cytometry analysis of cell surface staining of MHC class I in tumor cells after 6 days of co-culture of OVCAR-8 spheroids with PBMCs or tumor only. MHC class I-positive cells were gated. Percentage of MHC class I-positive population was labeled. (**b**) Flow cytometry analysis of cell surface staining of MHC class I in tumor spheroids treated with IFNγ of gradient concentration in the absence or presence of SHP099 and TNO155 for 6 days. MHC class I-positive cells were gated. Percentage of MHC class I-positive population was labeled. (**c**) MFI of MHC class I in tumor cells after 6 days co-culture of OVCAR-8-sg*AAVS1* or OVCAR-8-sg*IFNGR1* spheroids with PBMCs. Relative percentage of MFI of tumor MHC I (SHP099 treated over DMSO group) was labeled. (**d**) MFI fold change (stimulated/unstimulated) of MHC class I in tumor cells after 6 days co-culture of OVCAR-8-CAS9-sg*PTPN11*-1 or OVCAR-8-CAS9-sg*PTPN11*-2 spheroids with PBMCs (3 donors). OVCAR-8 cells were treated with or without doxycycline (100 ng/ml) for 5 days before co-culture. (**e**) Histogram of tumor cell surface PD-L1 level from 6 days of co-culture and tumor only. (**f**) Histogram of cell surface PD-L1 level in tumor spheroids treated with IFNγ of gradient concentration in the absence or presence of SHP099 (20 μM) and TNO155 (1 μM) for 6 days. (**g**) MFI of PD-L1 in tumor cells after 6 days co-culture of OVCAR-8-sg*AAVS1* or OVCAR-8-sg*IFNGR1* spheroids with PBMCs. Relative percentage of MFI of tumor PD-L1 (SHP099 treated over DMSO group) was labeled. (**h**) MFI of PD-L1 in tumor cells after 6 days co-culture of OVCAR-8-CAS9-sg*PTPN11*-1 or OVCAR-8-CAS9-sg*PTPN11*-2 spheroids with PBMCs (3 donors). OVCAR-8 cells were treated with or without doxycycline (100 ng/ml) for 5 days before co-culture. Flow cytometry data was analyzed and processed with FlowJo (Version 10.7.1, https://www.flowjo.com/solutions/flowjo/downloads/previous-versions).

ScRNAseq data revealed that antigen processing and presentation genes were upregulated by SHP099 treatment in tumor cells in co-culture (Fig. 4a). This was also true at the protein level as measured by FACS (Fig. 5a). In the context of rhIFNγ treatment of OVCAR-8 spheroids, HLA-ABC proteins were upregulated in a dose-dependent manner, and SHP2 inhibition substantially augmented their expression in cancer cells (Fig. 5b). Knockout of *IFNGR1* in OVCAR-8 cells mitigated the upregulation of MHC class I in co-culture upon SHP099 treatment (Fig. 5c and Supplementary Fig. 7a). Furthermore, genetic knockout of *PTPN11* in tumor cells enhanced MHC class I expression in co-culture, while the exogenous expression of the drug-resistant SHP2 mutant in tumor cells failed to upregulate tumor MHC class I levels upon SHP099 treatment (Fig. 5d and Supplementary Fig. 7b), thereby confirming that the enhanced IFNγ signaling in the cancer cells by SHP099 is due to on-target inhibition of SHP2. Defects in antigen presentation are associated with resistance to T cell-mediated tumor killing^35–37^, hence, upregulation of MHC class I expression on tumor cells by SHP2 blockade provides a rationale for combining SHP2 inhibition with immunotherapy in cancer patients.

Cancer cells exploit the immune inhibitory function of PD-L1 to evade the host immune system^38,39^. PD-L1 expression in cancer cells is positively regulated by T cell-derived IFNγ through the JAK-STAT signaling pathway^40^. We observed that PD-L1 expression was upregulated in tumor cells in co-culture with activated PBMCs, and that SHP2 inhibition further enhanced expression of PD-L1 (Fig. 5e and Supplementary Fig. 7c). SHP2 inhibition also boosted rhIFNγ-induced PD-L1 upregulation in OVCAR-8 spheroids (Fig. 5f and Supplementary Fig. 7d). The involvement of IFNγ signaling was confirmed by *IFNGR1* knockout in OVCAR-8 (Fig. 5g and Supplementary Fig. 7e). Similarly, knockout of *PTPN11* in tumor cells could increase PD-L1 expression in co-culture (Fig. 5h); in contrast, protecting tumor cells by overexpression of the SHP2 mutant impaired SHP099-induced PD-L1 upregulation (Supplementary Fig. 7f).

### SHP2 inhibition decreases tumor load in the 4T1 syngeneic mouse model

**Figure 6:**
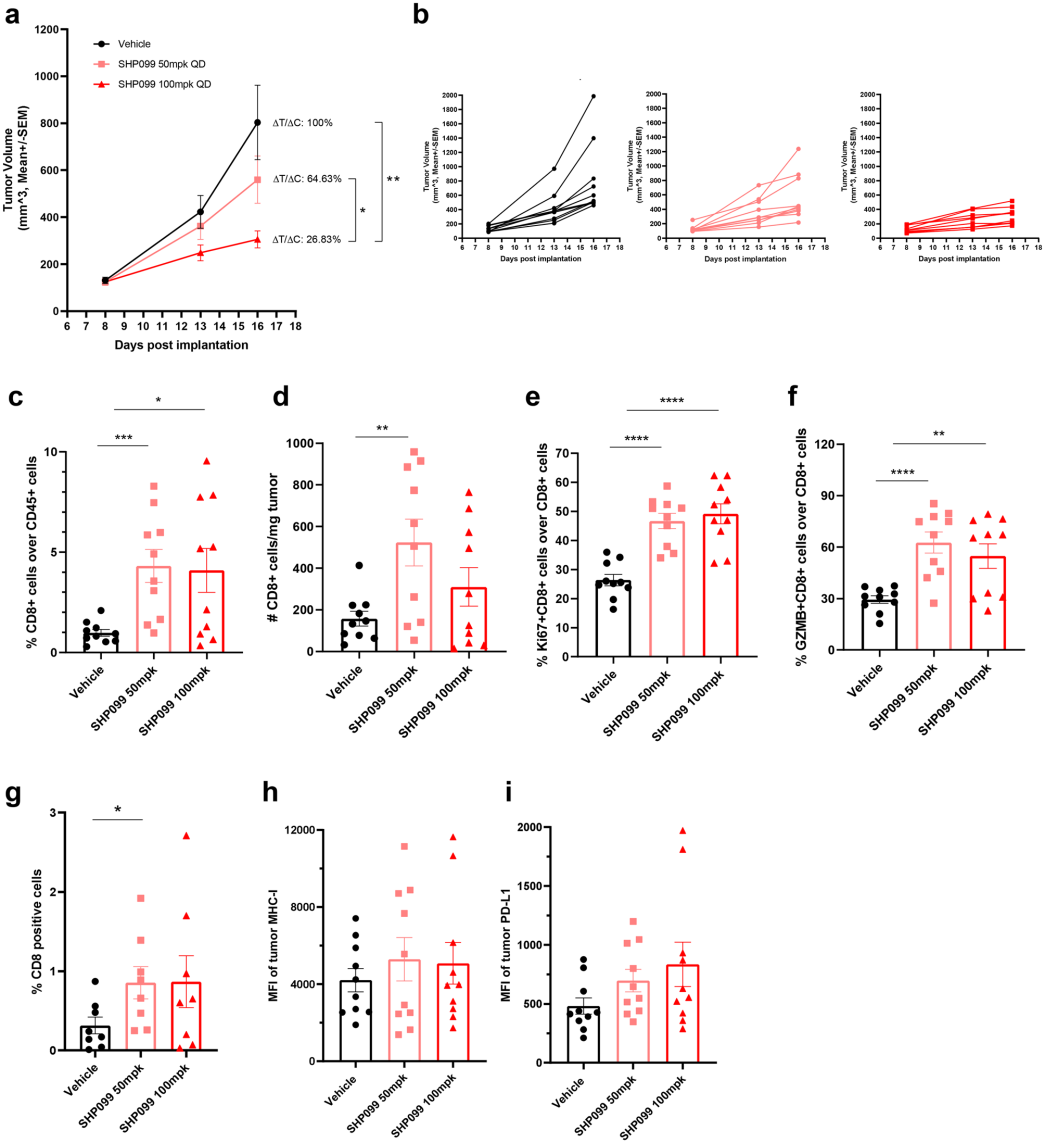
SHP2 inhibition decreases tumor load in the 4T1 syngeneic mice model. (**a**) Tumor growth curve (expressed as tumor volume) of subcutaneous 4T1 tumors in 4T1 syngeneic mice with different treatments. ΔT/ΔC of last time point was labeled. (**b**) Spider plot of tumor growth curve (expressed as tumor volume) of subcutaneous 4T1 tumors in 4T1 syngeneic mice with different treatments. (**c**) Flow cytometry analysis of the percentage of CD8+ T cells over CD45+ cells in tumor tissues with different treatments in 4T1 syngeneic model. (**d**) Flow cytometry analysis of the absolute number of CD8+ T cells per milligram tumor with different treatments in 4T1 syngeneic model. (**e**) Flow cytometry analysis of the percentage of Ki67+ CD8+ T cells over CD8+ cells in tumor tissues with different treatments in 4T1 syngeneic model. (**f**) Flow cytometry analysis of the percentage of GZMB+ CD8+ T cells over CD8+ cells in tumor tissues with different treatments in 4T1 syngeneic model. (**g**) IHC analysis of CD8 T cells in tumor tissues with different treatments from 4T1 syngeneic model. Y axis means the percentage of CD8+ cells over all the cells in the same slide. (**h**) MFI of the tumor surface MHC Class I with different treatments in 4T1 syngeneic model. (**i**) MFI of the tumor surface PD-L1 with different treatments in 4T1 syngeneic model. Flow cytometry data was analyzed and processed with FlowJo (Version 10.7.1, https://www.flowjo.com/solutions/flowjo/downloads/previous-versions).

To examine the effect of SHP2 inhibition on tumor immunity in vivo, we utilized the murine syngeneic 4T1 breast cancer model. Mice treated with SHP099 showed significantly attenuated tumor growth in a dose-dependent fashion (Fig. 6a,b). To understand if an intact immune system is necessary for this effect, we implanted 4T1 cells into immune-compromised NSG mice and treated with SHP099. SHP099 treatment led to a mild dose-dependent effect on 4T1 growth, indicating that SHP2 inhibition slows 4T1 tumor growth also in immune-compromised mice (Supplementary Fig. 8a, b). When SHP099 treatment data are normalized within each model by using the ΔT/ΔC formula, the higher dose of SHP099 treatment (100 mg/kg) in the 4T1 syngeneic model shows a considerably smaller ΔT/ΔC (26.83% vs. 50.01%), highlighting enhanced efficacy of SHP099 in the immune-competent 4T1 syngeneic model (Fig. 6a and Supplementary Fig. 8a). In summary, slower tumor growth in the 4T1 model caused by SHP099 treatment is likely to be at least partially attributable to enhanced anti-tumor immunity.

To investigate mechanisms of anti-tumor immunity triggered by SHP2 inhibition in vivo, we collected tumor tissue and performed immunohistochemistry and FACS analyses to profile immune phenotypes. We observed increased CD8 T cells in SHP099-treated tumors, which is consistent with SHP099-enhanced immune cell infiltration in co-culture (Fig. 6c,d). The percentage and number of CD4 T cells were unchanged by treatment (Supplementary Fig. 8c,d). In addition, SHP099 treatment significantly enhanced CD8 T cell proliferation and activation, also in line with in vitro data (Fig. 6e,f). IHC staining confirmed significant infiltration of CD8 T cells in 4T1 tumor tissues upon SHP099 treatment (Fig. 6g). To explore if IFNγ signaling was augmented in SHP099 treated tumors in vivo, we measured MHC Class I and PD-L1 expression. We observed an increased trend of expression at the protein level (Fig. 6h,i). At the transcriptional level, we observed significantly increased expression in SHP099 treated tumors at the lower dose (Supplementary Fig. 8e–g). Moreover, CXCR3 ligands *Cxcl9* and *Cxcl11* also showed a trend of enhanced mRNA expression in SHP099 treated groups (Supplementary Fig. 8h,i). To further illustrate the regulation of IFNγ signaling by SHP2 blockade in vivo, we utilized adoptive transfer of human bone Ewing’s sarcoma cells RD-ES and human PBMCs in NSG mice. SHP099 treatment upregulated expression of IFNγ pathway signature genes in RD-ES tumors specifically in the presence of human immune cells (Supplementary Fig. 8k–r).

Taken together, our in vitro and in vivo data demonstrates that SHP2 blockade enhances IFNγ signaling in tumors and triggers anti-tumor immunity via cytotoxic T cell recruitment and activation.

### SHP2 inhibition in cancer cells promotes T cell function in 4T1 syngeneic mouse model

**Figure 7:**
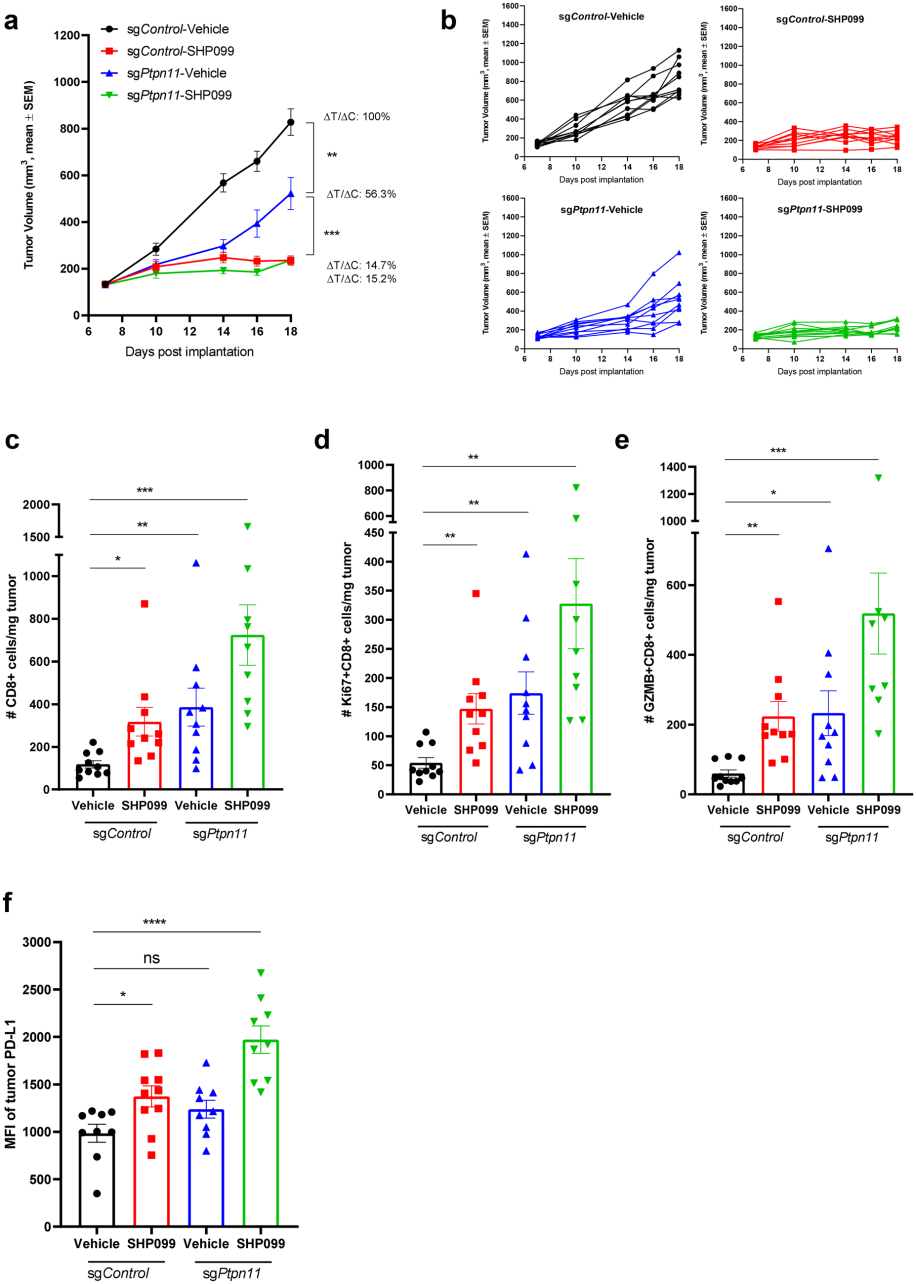
SHP2 inhibition in cancer cells promotes T cell function in 4T1 syngeneic mouse model. (**a**) Tumor growth curve (expressed as tumor volume) of subcutaneous 4T1-sg*Control* and 4T1-sg*Ptpn11* tumors in 4T1 syngeneic mice with different treatments. ΔT/ΔC of last time point was labeled. (**b**) Spider plot of tumor growth curve (expressed as tumor volume) of subcutaneous 4T1-sg*Control* and 4T1-sg*Ptpn11* tumors in 4T1 syngeneic mice with different treatments. (**c**) Flow cytometry analysis of the absolute number of CD8+ T cells per milligram tumor with different treatments in 4T1 syngeneic model. (**d**) Flow cytometry analysis of the absolute number of Ki67+ CD8+ T cells per milligram tumor with different treatments in 4T1-sg*Control* and 4T1-sg*Ptpn11* syngeneic model. (**e**) Flow cytometry analysis of the absolute number of GZMB+ CD8+ T cells per milligram tumor with different treatments in 4T1-sg*Control* and 4T1-sg*Ptpn11* syngeneic model. (**f**) MFI of the tumor surface PD-L1 with different treatments in 4T1-sg*Control* and 4T1-sg*Ptpn11* syngeneic model. Flow cytometry data was analyzed and processed with FlowJo (Version 10.7.1, https://www.flowjo.com/solutions/flowjo/downloads/previous-versions).

To understand the specific contribution of inhibiting SHP2 in the cancer cells to anti-tumor immunity in vivo, we knocked out SHP2 in 4T1 cells by direct electroporation of CAS9 protein and sgRNA targeting *Ptpn11* into 4T1 cells and established syngeneic model with 4T1-CAS9-sg*Ptpn11* cell pool which showed substantial knockout efficiency (Supplementary Fig. 9a). SHP2 knockout in cancer cells significantly slowed tumor growth, but not as efficiently as SHP099 treatment (100 mg/kg) (Fig. 7a,b). The ΔT/ΔC analysis indicated that targeting SHP2 in cancer cells induced less anti-tumor immunity than SHP099 (Fig. 7a), suggesting SHP099 mediated anti-tumor effects may also be mediated by SHP2 inhibition in immune or stromal cells.

Immune profiling analysis of tumor tissues illustrated that targeting SHP2 in 4T1 cells displayed increased CD8 T cell in tumors, which phenocopied the effect of SHP099 treatment (Fig. 7c). Consistent with in vitro data, SHP2 knockout in 4T1 cells promoted CD8 T cell proliferation and activation in 4T1 syngeneic model (Fig. 7d,e). Moreover, we observed an increased trend of PD-L1 expression in 4T1 cells when SHP2 gene was knocked out (Fig. 7f), suggesting that IFNγ signaling was enhanced by SHP2 depletion in tumor cells. The combination of tumor cell SHP2 knockout with SHP099 treatment exhibited more significant effect on regulation of T cell function and IFNγ signaling (Fig. 7c–f).

Overall, our in vivo genetic data confirmed our observation using pharmacological inhibition of SHP2, and further suggest that SHP2 inhibition in tumor cells can contribute to anti-tumor immunity. However, other inhibitory effect from SHP099 is required to decrease tumor load more efficiently in addition to targeting cancer cells.

### SHP2 blockade impairs the inhibitory effect of immunosuppressive myeloid cells

**Figure 8:**
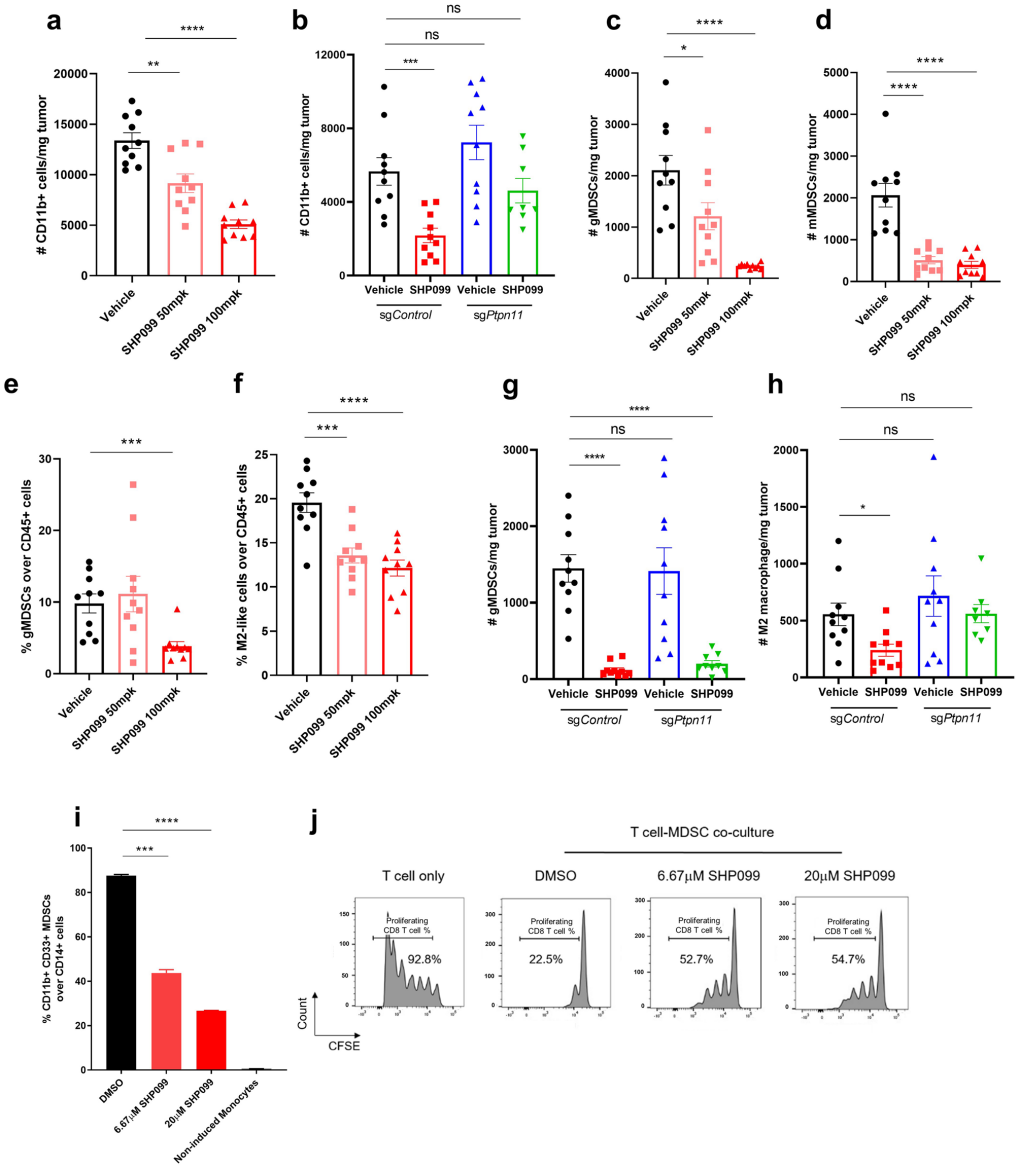
SHP2 blockade impairs the inhibitory effect of immunosuppressive myeloid cells. (**a**) Flow cytometry analysis of the absolute number of CD11b + myeloid cells per milligram tumor with different treatments in 4T1 syngeneic model. (**b**) Flow cytometry analysis of the absolute number of CD11b + myeloid cells per milligram tumor in 4T1-sg*Control* and 4T1-sg*Ptpn11* syngeneic model with different treatments. (**c**) Flow cytometry analysis of the absolute number of gMDSCs per milligram tumor with different treatments in 4T1 syngeneic model. (**d**) Flow cytometry analysis of the absolute number of mMDSCs per milligram tumor with different treatments in 4T1 syngeneic model. (**e**) Flow cytometry analysis of the percentage of gMDSCs over CD45+ cells in tumor tissues with different treatments in 4T1 syngeneic model. (**f**) Flow cytometry analysis of the percentage of M2-like macrophages over CD45+ cells in tumor tissues with different treatments in 4T1 syngeneic model. *(g*) Flow cytometry analysis of the absolute number of gMDSCs per milligram tumor in 4T1-sg*Control* and 4T1-sg*Ptpn11* syngeneic model with different treatments. (**h**) Flow cytometry analysis of the absolute number of M2-like macrophages per milligram tumor in 4T1-sg*Control* and 4T1-sg*Ptpn11* syngeneic model with different treatments. (**i**) Flow cytometry analysis of the percentage of CD11b+ CD33+ MDSCs over CD14+ monocytes after 5 days of GM-CSF and IL-6 induced MDSCs differentiation from monocytes with or without SHP099 treatment. (**j**) Flow cytometry analysis of the percentage of proliferating T cells after 6 days co-culture of differentiated MDSCs (treated with or without SHP099) with activated T cells (MDSCS:T cells = 1:2). T cells with diluted CFSE signal were gated. Percentage of T cells with diluted CFSE signal was labeled. Flow cytometry data was analyzed and processed with FlowJo (Version 10.7.1, https://www.flowjo.com/solutions/flowjo/downloads/previous-versions).

Differentiation of immature myeloid cells is often dysregulated in cancer, leading to the accumulation of immunosuppressive myeloid cells that promote cancer progression^41^. It has been reported that SHP2 inhibition produced a marked shift in polarized macrophage populations in tumor microenvironment in favor of anti-tumor immunity^42^. We observed a dose-dependent decrease of total CD45 + immune cells in SHP099-treated groups in the 4T1 syngeneic mouse model (Supplementary Fig. 9b). Further analysis indicated that much of this effect could be attributed to decreased CD11b + myeloid cells (Fig. 8a). Total numbers of CD3 + lymphoid cells were not affected by SHP099 treatment (Supplementary Fig. 9c). By using 4T1-sg*Ptpn11* syngeneic mouse model, we confirmed that the effect of SHP2 blockade on decreasing CD11b + myeloid cells was through direct targeting of myeloid lineage cells because SHP2 inhibition in tumor cells did not affect myeloid cells (Fig. 8b and Supplementary Fig. 9e,f).

We analyzed which subpopulations of tumor-infiltrated myeloid cells were affected by SHP099 treatment. Myeloid-derived suppressor cells (MDSC) are a heterogeneous group of immune cells that include monocytic (mMDSC) and granulocytic (gMDSC) subsets. Both of these cell populations accumulate in tumor-bearing mice and cancer patients and contribute to the immunosuppressive tumor microenvironment^43^. We observed a significant decrease in total numbers of gMDSCs and mMDSCs in SHP099-treated tumors, and a decrease in the percentage of gMDSCs in tumors treated with the higher dose of SHP099 (Fig. 8c–e and Supplementary Fig. 9f), suggesting that SHP2 inhibition affects the differentiation of MDSCs. Tumor-associated macrophages (TAMs) exist in the cancer microenvironment and influence tumor formation, growth, and metastasis^44^. Under diverse stimuli, macrophages can polarize and differentiate into cancer-inhibiting M1 and cancer-promoting M2 populations^45^. We discovered that SHP099 treatment significantly reduced the percentage of M2-like macrophages without affecting M1-macrophages (Fig. 8f and Supplementary Fig. 9g). Similarly, specifically knocking out SHP2 in tumor cells could not recapitulate SHP099-induced decrease of immunosuppressive gMDSCs and M2-like macrophages, implying a direct inhibitory effect of SHP099 on myeloid populations (Fig. 8g,h and Supplementary Fig. 9h).

To better understand the effect of SHP2 inhibition on MDSCs, we isolated CD14 + monocytes from whole blood of healthy donors and induced them to differentiate to immunosuppressive MDSCs by GM-CSF and IL-6 stimulation^41^; monocytes were treated with DMSO or SHP099 during differentiation. GM-CSF and IL-6 induced nearly 90% of cells to differentiate to an MDSC phenotype and SHP099 treatment dramatically inhibited differentiation in a dose-dependent way, suggesting that inhibiting SHP2 has a direct effect on myeloid differentiation (Fig. 8i). After 6 days, MDSCs were collected and co-cultured with CFSE-labeled activated T cells from the same donor at a ratio of 1:2 (MDSCs:T cells). T cell proliferation was measured by FACS after 5 days of co-culture. In control co-cultures, T cell proliferation was inhibited by MDSCs. This effect was partially reversed by treatment with SHP099 (Fig. 8j). These results indicate that SHP2 inhibition may enhance anti-tumor immunity by inhibiting the ability of monocytes to differentiate into MDSCs as well as by impairing the immune suppressive function of MDSCs.

### SHP2 inhibition displays combination benefit with PD1 blockade

**Figure 9:**
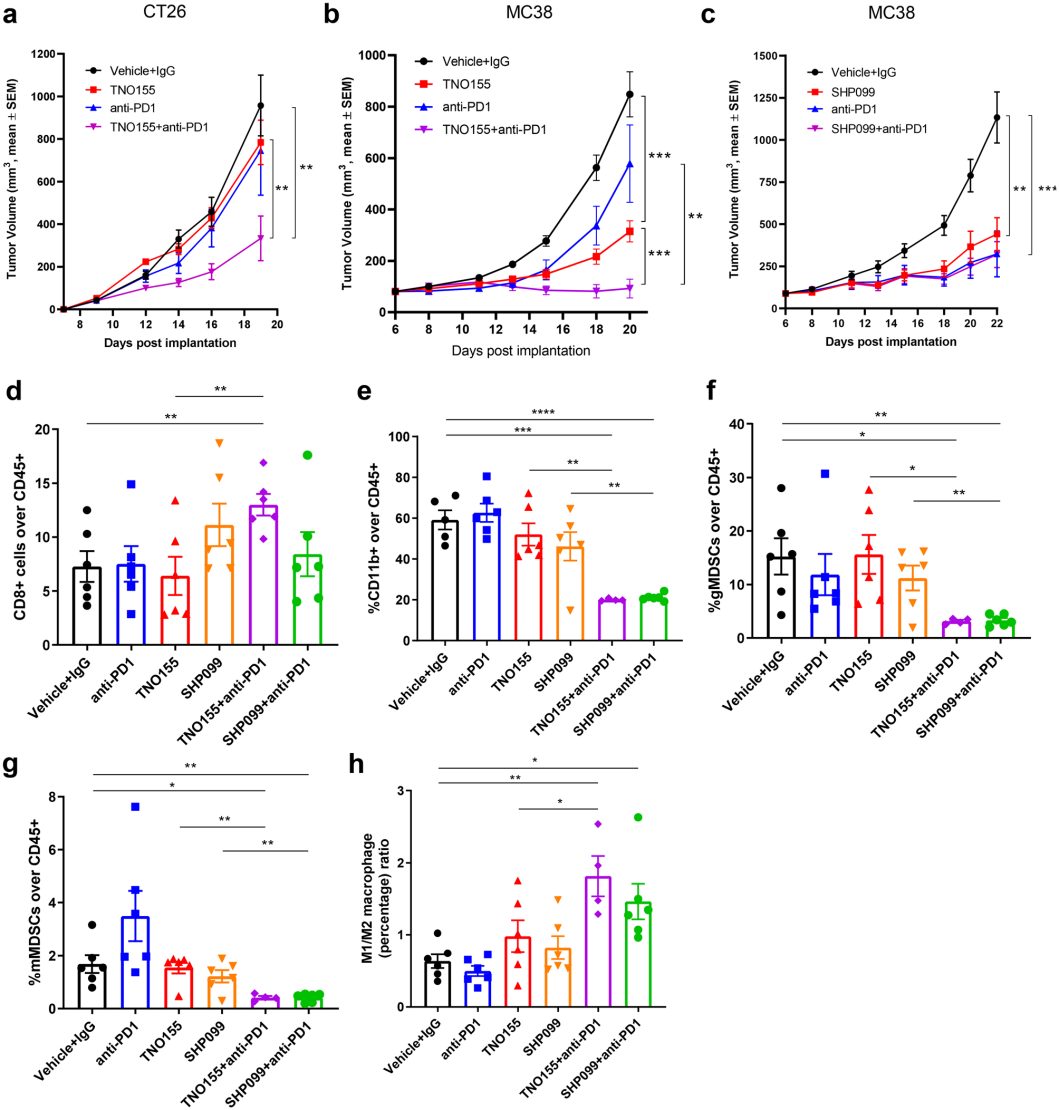
SHP2 inhibition displays combination benefit with PD1 blockade. (**a**) Tumor growth curve (expressed as tumor volume) of subcutaneous CT26 tumors in CT26 syngeneic mice with different treatments. TNO155: 20 mg/kg, PO, BID; Anti-PD1: 10 mg/kg, IP, QW. (**b**, **c**) Tumor growth curve (expressed as tumor volume) of subcutaneous MC38 tumors in MC38 syngeneic mice with different treatments. Mice were treated with Vehicle + IgG, anti-PD1, TNO155 and TNO155 + anti-PD1 in (**b**); Mice were treated with Vehicle + IgG, anti-PD1, SHP099 and SHP099 + anti-PD1 in (**c**); TNO155: 20 mg/kg, PO, BID; SHP099: 100 mg/kg, PO, QD; Anti-PD1: 10 mg/kg, IP, QW. (**d**) Flow cytometry analysis of the percentage of CD8+ T cells over CD45+ immune cells in MC-38 syngeneic model. (**e**) Flow cytometry analysis of the percentage of CD11b+ myeloid cells over CD45+ cells in MC-38 syngeneic model. (**f**) Flow cytometry analysis of the percentage of gMDSCs over CD45+ cells in MC-38 syngeneic model. (**g**) Flow cytometry analysis of the percentage of mMDSCs over CD45+ cells in MC-38 syngeneic model. (**h**) Flow cytometry analysis of the percentage ratio of M1 macrophage over M2 macrophage in MC-38 syngeneic model. Flow cytometry data was analyzed and processed with FlowJo (Version 10.7.1, https://www.flowjo.com/solutions/flowjo/downloads/previous-versions).

Clinically, high tumor expression of PD-L1 is associated with tumor immune escape and poor prognosis^46–48^. Immune checkpoint inhibition, particularly PD1 blockade, has revolutionized the clinical oncology practice^49–51^. We showed upregulated tumor PD-L1 expression following SHP2 inhibition both in vitro and in vivo, which could be a potential barrier to the efficacy of this treatment. Combination of anti-human PD1 antibody with SHP2 inhibitors showed combinatorial benefit on enhancing immune cells-mediated tumor killing in co-culture of OVCAR-8 spheroids with activated PBMCs, while PD1 antibody did not show any single-agent effect (Supplementary Fig. 10a,b). Blockade of SHP2 in combination with anti-PD1 synergistically attenuated tumor growth in the murine syngeneic CT26 colon carcinoma model (Fig. 9a). Combination benefit was also observed in the MC38 colorectal cancer model (Fig. 9b), although, there is variation of the single-agent anti-tumor effect of anti-PD1 (Fig. 9c and Supplementary Fig. 10d). SHP2 blockade displayed single-agent effect on tumor growth inhibition since MC38 syngeneic mice treated with SHP2 inhibitors (TNO155 or SHP099) had significantly smaller tumors in comparison to control groups (Fig. 9b,c and Supplementary Fig. 10d). MC38 tumor cells are insensitive to direct inhibition of SHP2 as they carry the G503V mutation of *Ptpn11* gene. We confirmed this by treating MC38 tumors with SHP099 and measuring expression of Dusp6, a gene positively regulated by SHP2 (Supplementary Fig. 10c). Indeed, SHP099/TNO155 treatment did not significantly inhibit MC38 xenograft growth in immune-compromised NSG mice (Supplementary Fig. 10e,f), suggesting that SHP2 inhibition in immune cells contributed to decreasing the tumor load in MC38 syngeneic mouse model.

Next, we profiled tumor-infiltrated immune cells from MC38 tumors. Similarly to what was observed in the 4T1 model, tumor-infiltrated total CD45 + cells significantly decreased after SHP099/TNO155 treatment, while combination with PD1 antibody further decreased the percentage (Supplementary Fig. 10g). We also observed that these treatments did not affect the number of CD4 + T cells but increased the percentage of CD8 + T cells significantly only in the TNO155 plus anti-PD1 combination arm (Fig. 9d and Supplementary Fig. 10h). Unlike in the 4T1 model, SHP2 inhibition alone did not enhance tumor infiltrated CD8+ T cells in MC38 syngeneic model (Fig. 9d), which suggests that targeting tumor intrinsic signaling contributed to CD8+ T cell recruitment to the tumor microenvironment, in consideration that MC38 is SHP2 inhibitor-insensitive. Blockade of SHP2 and PD1 showed combinatorial benefit on decreasing CD11b+ myeloid cells (Fig. 9e), which might contribute to decreased CD45+ immune cells in tumors. Percentages of both immunosuppressive myeloid-derived gMDSCs and mMDSCs showed a strong decrease after SHP099/TNO155 treatment in combination with immune checkpoint blockade (Fig. 9f,g). The combination also effectively increased the ratio of immune-promoting M1 macrophages over immunosuppressive M2 macrophages (Fig. 9h).

Taken together, these data indicate that SHP2 inhibition in combination with immune checkpoint blockade boosts anti-tumor immunity through targeting immunosuppressive cells of the myeloid lineage in the tumor microenvironment, potentially leading to enhanced T cell-mediated tumor cell killing.

## Discussion

Inhibition of the phosphatase SHP2 exhibits therapeutic potential in cancers dependent on receptor tyrosine kinases and mutated KRAS signaling, as SHP2 plays a critical role in mediating MAPK signaling in cancer cells^15,16^. In this study, by utilizing both in vitro co-cultures of tumor spheroids and human immune cells and in vivo syngeneic mouse models, we discovered that inhibiting SHP2 activity alters the cellular composition of the tumor microenvironment through its effect on both tumor and immune cell populations to promote anti-tumor immunity.

Specifically, SHP2 inhibition enhanced T cell-mediated tumor killing and T cell proliferation/activation in vitro. Mechanistically, SHP099/TNO155 induced these effects through targeting SHP2 in cancer cells and augmenting cancer cell IFNγ signaling in the context of tumor-immune cell crosstalk. In vivo, SHP2 inhibition also increased tumor IFNγ signaling and displayed anti-tumor activity by promoting cytotoxic T cell function and inhibiting immune suppressive myeloid cells.

In cancer cells, SHP2 inhibition potentiated the response to IFNγ by enhancing JAK-STAT signaling. Consequently, IFNγ pathway targets including CXCL9, -10, and -11, MHC Class I, and PD-L1, were upregulated at both the transcriptional and protein levels. Defects in IFNγ signaling cause resistance to T cell-mediated tumor killing in both pre-clinical models and cancer patients^35,52,53^. In this study, we demonstrated the importance of IFNγ signaling in our tumor model by knocking out the IFNγ receptor, IFNGR1; we observed impaired T cell-mediated tumor killing as well as attenuated MHC class I expression in co-culture. PD-L1 expressed on the surface of cancer cells serves as a negative feedback mechanism to anti-tumor immunity. Clinically, higher PD-L1 expression in the tumor microenvironment has been positively correlated with response to immune checkpoint blockade with PD1 antagonists^38,39^. Since SHP2 inhibition augmented PD-L1 expression in cancer cells in co-culture with T cells, we combined SHP2 blockade with immune checkpoint inhibition and the combo showed combinatorial benefit on boosting anti-tumor immunity and slowing down tumor growth. This raises the possibility that therapeutic inhibition of SHP2 could be effectively combined with checkpoint blockade to enhance treatment efficacy in clinic.

Our in vitro MDSC differentiation and T cell-MDSC co-culture experiments showed that SHP2 inhibition could directly affect myeloid cells by inhibiting their immunosuppressive function. We also observed modulation of myeloid cell subsets by SHP2 inhibition in both the 4T1 and MC38 syngeneic mouse models. The presence of immunosuppressive M2 macrophages and MDSCs in the tumor microenvironment was decreased by SHP2 inhibition. From these observations we hypothesize that modulation of myeloid cells may contribute to tumor growth inhibition by SHP099/TNO155 treatment in vivo.

Taken together, our data show that SHP2 inhibition is effective in controlling tumor growth by enhancing immune surveillance and cancer cell killing. We propose that this is achieved by: (a) amplifying IFNγ signaling in cancer cells^54^; (b) enhancing chemoattractant cytokine secretion by cancer cells which may recruit anti-tumor lymphocytes to the tumor; (c) increasing antigen presentation by cancer cells; (d) positively regulating CD8 T cell proliferation and function; (e) inhibiting the function of immunosuppressive myeloid cells on anti-tumor T cells.

In light of published work and the data presented in this study, we propose that inhibition of SHP2 activity in cancer patients has the potential to be therapeutically beneficial by both directly inhibiting cancer cell growth and promoting anti-tumor immunity.

## Materials and methods

### Cell lines and compound

Cell lines were obtained from Novartis’ CCLE collection^55^ and were tested to be free of mycoplasma. OVCAR-8, OV-90, RD-ES, DMS-273 and 4T1 cells were cultured in RPMI1640 media (Gibco, Thermo Fisher Scientific) containing 10% fetal bovine serum (FBS, GE Life Sciences). HEK293T, MIA PaCa-2 and MC-38 cells were cultured in DMEM media (Gibco, Thermo Fisher Scientific) containing 10% FBS. All cells were maintained in a humidified incubator at 37 °C with 5% CO_2_. For co-culture with immune cells, media of cells cultured in DMEM were changed to RPMI1640 media before seeding into Elplasia plates. SHP2 inhibitor SHP099 and TNO155 was synthesized and structurally verified by NMR/LC–MS at Novartis Institutes for BioMedical Research. Recombinant human IFNγ (#285-IF-100) was purchased from R&D Systems. Murine anti-PD1 clone 332.1D2 is a kind gift of Prof. Gordon Freeman at DFCI. Human anti-PD1 is produced and verified by Novartis Institutes for BioMedical Research.

### Co-culture of tumor spheroids and PBMCs

Elplasia square type 384-well-plates (SQ 200 100 384, Kuraray), which have 225 100 × 200 μm micro-spaces divided by wall in each single well, were coated with poly-2-hydroxyethyl methacrylate (poly-HEMA) (192066, Sigma-Aldrich). As Supplementary Fig. 1a shows, 2 × 10^4^ tumor cells were seeded per well on Day-1 and cultured overnight to form round shaped spheroids. Freshly isolated human PBMCs from healthy donor blood were seeded on top of the spheroids at E-to-T ratio of 1:1 on the following day (Day0). PBMCs were either unstimulated or stimulated with Dynabeads CD3/CD28 Human T-Activator (11132D, Thermo Fisher Scientific) at bead-to-cell ratio of 1:8. The co-culture system was treated with compounds on Day0 as well after PBMCs seeding and then underwent co-culture for days. Multiple donors were used in co-culture with unknown HLA (human leukocyte antigen) matching status. RPMI1640 media containing 10% FBS was used for co-culture. Cells with doxycycline-inducible sgRNA or SHP2-WT/Mutant were treated with 100 ng/ml doxycycline for 5 days before co-culture. ACCUMAX cell detachment solution (#7921, STEMCELL Technologies) was used to dissociate tumor spheroids into single cells for flow cytometry analysis.

Human peripheral blood mononuclear cells (PBMCs) were isolated by spinning CPT tubes (BD Bioscience #362761) containing exsanguinated whole blood at 1800 rpm for 20 min and slowing down without brake. All studies with human blood were performed under ethical approval by the WCG Clinical Western Institutional Review Board (WIRB)-Copernicus Group and in compliance with the guideline from Standard Operating Procedure for Obtaining Venous Blood and Other Non-Invasive Biological Specimens for the Novartis Institutes for Biomedical Research, Inc. All the participants provided informed consent prior the study.

### Light sheet fluorescence microscopy

5000 OVCAR-8 cells constitutively expressing mCherry were seeded in each well of a 384 ULA round bottom well plate and let form spheroids overnight. CFSE-labeled PBMCs were seeded on the following day for co-culture and treated with SHP099. After 24 h of treatment, spheroids were imaged using a Zeiss Lighsheet Z.1 microscope. Each spheroid was embedded in 2% agarose in 1 × HBSS and drawn into a 1 mm glass capillary tube. The capillary tube was placed into the imaging chamber that was filled with PBS. The imaging objective was a 20X, 1.0 NA water immersion with 10X illumination objectives. The spheroids were centered in the field of view (584X584 μm) and a z-stack (0.5 μm steps) with two fluorescence channels was collected with channel 1 settings 488 nm laser (1.5% power), emission filter 505–545 nm (15 ms exposure) and channel 2 settings 561 nm laser (0.5% poser), emission filter 575–615 nm (15 ms exposure). The illumination was set to dual side to increase depth of imaging and dual side image data was fused using maximum intensity fusion.

### Single-cell RNA sequencing

Single cell libraries were prepared according to 10X Genomics specifications (Chromium Single Cell V(D)J User Guide PN-1000006, and the v2 Reagent Kit (PN-10000075, PN-1000153). The quality of the cDNA was assessed using an Agilent TapeStation 4200, High Sensitivity D5000 Kit (5067–5592, 5067–5593) and the quality of the libraries were assessed using the High Sensitivity D1000 Kit (5067–5584, 5067–5585). Libraries were diluted to 10 nM and clustered using Illumina’s MiSeq, on a 150-cycle v3 paired-end read flow cell and sequenced for 26 cycles on R1 (10X barcode and the UMIs), followed by 8 cycles of I7 Index (sample Index), and 98 bases on R2 (transcript), in order to normalize for read depth and cell number. Libraries were then clustered and sequenced using Illumina’s HiSeq4000 on a paired-end flow cell, achieving a sequencing depth of around 50,000 reads per cell.

Raw sequencing data were processed using cellranger v3.0 from 10X Genomics to generate sequencing and alignment QC metrics and the gene by cell unique molecular identifiers (UMI) count matrix. The unfiltered raw output from Cellranger was loaded with Scanpy^56^ and filtering was applied to account for cells with a minimum number of expressed genes (300) and genes expressed in a minimum number of cells. After normalization, further filtering was applied to keep genes with a minimum average expression of 0.0125 (log10) and dispersion greater than 0. We performed dimensionality reduction taking into account 40 principal components and clustered the cells using the Louvain algorithm^57^. The population of cells was then divided into two groups: tumor and immune, based on the CD45 expression of each Louvain cluster (Supplementary Fig. 4a). Those populations were later analyzed independently to estimate gene expression levels for Figs. 3a and 4a. The immune cells were divided into 5 major groups based on different gene expression sets of immune populations (Supplementary Fig. 4b). The pathway signature analysis is conducted in https://metascape.org/gp/index.html#/main/step1. IFNγ signaling pathway signature genes are from GSEA/mSigDB hallmark gene set collection (http://software.broadinstitute.org/gsea/msigdb/collections.jsp#H).

### In vivo mouse models

All animal studies were performed under approval by the Novartis Institutes for BioMedical Research Institutional Animal Care and Use Committee and in compliance with the Guide for the Care and Use of Laboratory Animals. The methods were adapted/adjusted from the methods of another literature published by Novartis^58^: before implantation, all cell lines were confirmed as mycoplasma- and rodent virus-negative. 0.25 × 10^6^ 4T1 cells in Hank’s balanced salt solution (HBSS) were inoculated subcutaneously into the right flank of BALB/c mice (for immune-competent syngeneic mouse model) or NSG mice (for immune-compromised xenograft model). 1 × 10^6^ MC-38 cells in HBSS were inoculated subcutaneously into the right flank of C57BL/6 mice (for immune-competent syngeneic mouse model) or NSG mice (for immune-compromised xenograft model). Tumor bearing mice were randomized into 3 treatment groups once tumor volumes reached 100 ~ 150 mm^3^. 0.2 × 10^6^ CT26 cells in HBSS were inoculated subcutaneously into the right flank of C57BL/6 mice (for immune-competent syngeneic mouse model). Generally on Day 6–9 after inoculation, tumor bearing mice started to receive treatments. Small molecule compound treatments were dosed via oral gavage. Anti-PD1 treatment was dosed via intraperitoneal injection. Treatment groups includes: Vehicle (0.5% MC/0.5% Tween 80, daily), SHP099 (50 mg/kg, daily), SHP099 (100 mg/kg, daily), TNO155 (20 mg/kg, twice per day), anti-mouse IgG (10 mg/kg, weekly), anti-mouse PD1 (10 mg/kg, weekly). Tumors were measured twice weekly by calipering in two dimensions. Tumor volume was calculated using a modified ellipsoid formula: tumor volume = L × W^2^ × π/6, where L is the longest axis of the tumor and W is perpendicular to L. Anti-tumor activity was reported as percentage treatment/control (%T/C) values and calculated using the formula %T/C = 100 × (ΔT/ΔC); here ΔT is the mean tumor volume (mTV) of the SHP099-treated group on day f (final day) minus the mTV of the SHP099-treated group on day i (initial dosing day), and ΔC is the mTV of the Vehicle group on day f minus the mTV of the Vehicle group on day i. Syngeneic mice for PD study were euthanized on Day 7 after dosing and tumor tissues were taken for digestion and immunostaining.

### Tumor tissue digestion

For tumors from 4T1 syngeneic mice, the digestion was conducted following reference^59^: tumor tissues were minced into fine pieces (approximately 1 mm^3^) using scissors and razor blades, transferred into 15 mL conical tubes containing 2 ml of digestion buffer [RPMI1640 (Gibco, Thermo Fisher Scientific), 2% FBS, 0.2 mg/ml Collagenase P (#11249002001, Roche), 0.2 mg/ml Dispase (#17105-041, Gibco), and 0.1 mg/ml DNase I (#10104159001, Roche)], and placed into a water bath at 37 °C. The tubes were vortexed every 5 min for 15 min, the tissue pieces were allowed to settle for 5 min, and then the supernatant containing freed cells was collected and quenched at 4 °C in 50 mL conical tubes containing 20 ml of cold flow cytometry buffer (PBS, 2% FBS, and 2 mmol/l EDTA). Next, 2 ml of fresh digestion buffer was added to the remaining tumor fragments, and tubes were incubated at 37 °C for another 20 min, vortexing every 5 min, prior to collecting the freed cells and adding them to the previously collected fractions on ice. These 20 min digestion cycles were repeated for a total of 5 to 6 times, with progressively more forceful agitation methods (vortexing, pipetting 1 ml up and down using large orifice tips, then mixing with uncut 1 ml tips), until no tumor fragments larger than 1 mm remained. The collection tube containing the digested fractions in flow cytometry buffer was kept on ice until the digestion was complete, and then the contents was filtered through 70 μm mesh, centrifuged (1500 rpm, 10 min, 4 °C), and the cells were counted. Pellets were resuspended in flow cytometry buffer and subjected to immunostaining. For Q-PCR, pellets went through CD45, CD90 and CD31 positive enrichment to remove immune cells and stromal cells by using Easysep Biotin Positive Selection Kit (#18559, STEMCELL Technologies) following the manufacturer’s instruction. Negative portion (tumor cells) was collected for future Q-PCR analysis. Antibodies used for positive enrichment are: Biotinylated CD45 (#103104, Biolegend), Biotinylated CD31 (#102504, Biolegend), Biotinylated CD90/Thy1.2 (13-0902-85, Invitrogen).

For tumors from MC-38 syngeneic mice, tumor tissues were put into gentleMACS C tubes (MACS Miltenyi Biotec) containing 2 ml RPMI1640 and minced into fine pieces (approximately 1 mm^3^) using scissors. Another 7 ml warmed RPMI1640 and 1 ml Liberase Thermolysin Medium/DNase I stock solution [0.17 mg/ml Liberase Thermolysin Medium (#5401127001, Roche), 14 U/ml DNase I (#4716728001, Roche)] were added into C tubes. Tightly closed C tubes were attached upside down onto the sleeve of the gentleMACS Dissociator and ran through Program h_impTumor_01. Next, C tubes were incubated at 37 °C for 5 min and quenched with 1:10 dilution of FBS. Then program h_impTumor_02 was ran twice on C tubes. After termination of the program, tumor tissue cell suspensions were applied to 70 μm cell strainers placed on 50 ml conical tubes. The collection tubes containing the suspensions were centrifuged (1500 rpm, 10 min, 4 °C) and the cells were counted. Pellets were resuspended in flow cytometry buffer and subjected to immunostaining.

### Electroporation in 4T1 murine breast cancer cells

Electroporation of sg*Ptpn11*, tracrRNA (#1075928, IDT) and CAS9 protein (A36498, Invitrogen) into 4T1 cells to establish 4T1-CAS9-sg*Ptpn11* pool was performed following the manufacturer’s instruction by using SE Cell Line 4D-Nucleofector X Kit S (#V4XC-1032, Lonza) and Nucleofector 4D, X-unit (#AAF-1002X, Lonza). CM-150 electroporation program was used for 4T1 cells. sg*Control* (#1072544, IDT) was used as negative control. sg*Ptpn11* target sequence is listed below. Ablation of target gene was validated by western blots (Supplementary Fig. 9a).

sg*Ptpn11*: GCTTGCTTAACTCTCGAACC.

### In vitro generation of human MDSCs and suppression assay

Human PBMCs were isolated from healthy volunteer donors by density gradient separation (#17144003, GE Life Sciences). Monocytes were isolated from PBMCs by positive selection using anti-CD14 magnetic microbeads (#130-050-201, Miltenyi Biotec) and LS column separation (#130-042-401, Miltenyi Biotec), per manufacturer’s instructions. Monocytes were cultured at 5 × 10^5^ cells/ml in RPMI1640 containing 10%FBS, supplemented with recombinant human GM-CSF (10 ng/ml, Peprotech) and IL-6 (10 ng/ml, Peprotech) in the presence or absence of SHP099 for 6 days in humidified 5% CO_2_ incubator. After 6 days of differentiation, adherent MDSCs were detached using EDTA solution (5 mM, Invitrogen). MDSCs were quantified and co-cultured with autologous T cells as mentioned below. Autologous T cells were isolated from PBMCs by immunomagnetic negative selection (#17951, STEMCELL Technologies), and frozen in cryopreservation medium with 10% DMSO (CryoStor CS10, STEMCELL Technologies). The day before co-culture with autologous monocyte-derived MDSCs, T cells were thawed in RPMI media containing 10% FBS and recombinant human IL-2 (5 ng/ml, NIBR) and rested overnight in an incubator (5% CO_2_). The day of the co-culture, T cells were labelled with CFSE (#423801, Biolegend) and quantified for co-culture with MDSCs at seeding densities of MDSC:T = 1:2 in triplicates in round bottom 96 well culture plate (M9436-100EA, Greiner Bio-One). The co-culture media comprised of Gibco RPMI1640 containing 10% FBS, recombinant human IL-2 (5 ng/ml, NIBR) and soluble human anti-CD3/CD28 antibody (#10971, STEMCELL Technologies). Undifferentiated monocytes were used as mock-MDSC controls. After 5 days of MDSC/T cell co-culture, cells were analyzed by flow cytometry to measure level of suppression by MDSCs by measuring T cell proliferation as a function of dilution of CFSE dye. MDSCs on day of harvest and 5 days post co-culture with T cells were evaluated for CD14, CD11b and CD33. Samples were run on LSR-II, BD Pharmingen for flow cytometry and analyzed using FlowJo software (v10.6.2, BD Biosciences), and data plotted using graphpad prism software. The following antibodies were used, CD14 (#563561, BD Horizon), CD11b (#301322, Biolegend), CD33 (#303430, Biolegend), CD8 (#344748, Biolegend).

### Gene ablation using CRISPR-CAS9 system

Cells stably expressing CAS9 were generated as previously described^58^ and infected with lentivirus expressing sgRNA in the presence of 8 μg/ml Polybrene for 24 h. Because RFP was co-expressed with sgRNA from the same construct, RFP-positive cell population was sorted by flow cytometry. sgRNA target sequences used in this work are listed below. Ablation of target genes was validated by western blots or flow cytometry (Supplementary Figs. 2f and 6e).

- sg*PTPN11*-1: TGCGCACTGGTGATGACAAA; sg*PTPN11*-2: GACCACGGCGTGCCCAGCGA;
- sg*AAVS1*: GGGGCCACTAGGGACAGGA; sg*IFNGR1*: AGATGGGCACCGCGGATCTG;
- sg*B2M*: GAAGTTGACTTACTGAAGAA.

### Luminex and ELISA

Luminex detection of a panel of cytokines was performed following the manufacturer’s instruction by using Human Cytokine/Chemokine Magnetic Bead Panel, 96 Well Plate Assay (#HCYTOMAG-60K-PX22, EMD Millipore). ELISA detection of CXCL10 and IFNγ level was performed following the manufacturers’ instruction by using CXCL10 (Human) ELISA kit (KA2004, Abnova) and Human IFN-gamma Quantikine ELISA kit (DIF50, R&D Systems), respectively.

### Flow cytometry analysis

Single cell suspensions were resuspended in flow cytometry buffer (PBS, 2% FBS, and 2 mmol/l EDTA), blocked with Fc block, and then stained with different fluorochrome-conjugated antibodies for 30 min on ice. For intracellular staining, cells were fixed and permeabilized with FOXP3/Transcription Factor staining buffer set (00-5523-00, Invitrogen, Thermo Fisher Scientific). For cell counting, CountBright absolute counting beads (C36950, Invitrogen, Thermo Fisher Scientific) were added and absolute cell number was calculated according to the manufacturer’s instruction. Samples were run on BD LSRFortessa Cell Analyzer and analyzed with FlowJo (Version 10.7.1). License for FlowJo was purchased via the link https://www.flowjo.com/solutions/flowjo/site-licensing. Antibodies used to stain human samples include: anti-CD45 (#304016, Biolegend), anti-GZMB (#372204, Biolegend), anti-CXCL10 (#519504, Biolegend), anti-IFNγ (#502512, Biolegend), anti-HLA-A,B,C (#311426, Biolegend), anti-PD-L1 (#374514, Biolegend), anti-CD8a (#300908, Biolegend), anti-CD3 (#300408, Biolegend), anti-CD14 (#325608, Biolegend); anti-IFNGR1 (MCA1450A647, Bio-Rad), Viability dye (L23102, L34975, Thermo Fisher Scientific); CFSE Cell Division Tracker kit (#423801, Biolegend) was used to label immune cells. Antibodies used to stain mouse samples include: anti-CD45 (#103151, Biolegend), anti-H-2 Kb, H-2Db (#114614, Biolegend), anti-PD-L1 (#124314, Biolegend), anti-CD90.2 (#564365, BD Bioscience), anti-CD31 (#612802, BD Bioscience), anti-CD11b (#564443, BD Bioscience), anti-CD3 (#564010, BD Bioscience), anti-CD4 (#100447, Biolegend), anti-CD8a (#100722, Biolegend), anti-Foxp3 (#126404, Biolegend), anti-GZMB (#372204, Biolegend), anti-Ki67 (#561284, BD Bioscience), anti-Ly6G (#127643, Biolegend), anti-Ly6C (#128018, Biolegend), anti-F4/80 (#123141, Biolegend), anti-CD11c (#117310, Biolegend), anti-MHC-II (I-S/I-E) (#107606, Biolegend), anti-CD103 (#121406, Biolegend); Viability dye (L34975, Thermo Fisher Scientific).

### Immunohistochemistry (IHC)

Freshly isolated tumor tissues were immediately fixed in formaldehyde solution for 24 h and then put in 70% ethanol before immunostaining. IHC was run on Roche Ventana Discovery Ultra automatic platform. The reagents used in IHC are: DISC ChromoMap DAB RUO (Detection kits, #760-159, Ventana), DISC OmniMap anti-Rb HRP RUO (Secondary Antibody, #760-4311, Ventana), DISC Hematoxylin (#760-2021, Ventana), DISC Bluing reagent (#760-2037, Ventana), CD8α (D4W2Z) XP Rabbit mAb (Mouse Specific), (Primary Antibody, #98941, Cell Signaling Technology).

### Lentivirus packaging

Early-passage HEK293T cells were plated at a density of 2.5 × 10^6^ per BD BioCoat Collagen I 100 mm Culture Dish (BD Biosciences) 24 h before transfection. Cells were transfected with 1.7 µg ready-to-use lentiviral packaging plasmid mix (#CPCP-K2A, CELLECTA) with 1.4 µg of sgRNA or SHP2-WT/MUT construct in Optimem Serum Free Medium (#11058021, Invitrogen) using the TransIT-293 transfection reagent (# MIR 2700, Mirus). Medium was replaced at 18 h after transfection, and viral supernatants were harvested at 48 h after medium replacement. Viral supernatants were filtered through 0.45 µm cellulose acetate filters (Corning).

### Exogenous expression of wildtype SHP2 and mutant SHP2

Cells exogenously expressing either wildtype SHP2 or mutant SHP2 were generated by infection with lentivirus packaged using the pLKO-Trex-SBP-SHP2-WT or pLKO-Trex-SBP-SHP2-T253M/Q257L plasmids in the presence of 8 μg/ml Polybrene for 24 h. Cells were selected in TET-free medium containing 1 mg/ml G418.

### RNA isolation, cDNA synthesis and quantitative PCR

RNA isolation for mouse tumors was performed with the QIAGEN QIAcube and RNeasy Plus Mini Kit (#74134, QIAGEN). Total RNA (1 μg) isolated from tumors was used to synthesize cDNA with a High-Capacity cDNA Reverse Transcription Kit (#4368814, Thermo Fisher Scientific). Quantitative PCR was performed using Taqman Fast Advanced Master Mix (#4444554, Thermo Fisher Scientific) and probe-based assays: Cxcl9, Cxcl10, Cxcl11, Cd274 (PD-L1), H2-k1, H2-d1, Dusp6, Actin (Integrated DNA Technologies). The relative expression levels of genes were calculated with the 2^−ΔΔCt^ method.

### Western blot

Western blots were performed following the method from another literature published by Novartis^58^: total cell lysates were prepared by direct lysis of cells with RIPA buffer (Thermo Fisher Scientific) containing protease and phosphatase inhibitors (Thermo Fisher Scientific) with incubation on ice for 20 min and followed by centrifugation at 12,000×*g* at 4 °C for 5 min. The supernatant was collected and protein concentration was determined using Pierce BCA protein assay kit (#23225, Thermo Fisher Scientific). Equal amounts of proteins were resolved by SDS-PAGE, transferred to polyvinylidene difluoride (PVDF) membranes and probed by the following antibodies with indicated dilutions: phospho-ERK1/2 (T202/Y204) (9101, Cell Signaling Technology, 1:1000), total ERK1/2 (9102, Cell Signaling Technology, 1:1000), SHP2 (3397, Cell Signaling Technology, 1:1000), phospho-STAT1 (Y701) (9167, Cell Signaling Technology, 1:1000), total STAT1 (14994, Cell Signaling Technology, 1:1000), Vinculin (13901, Cell Signaling Technology, 1:1000). After detection of phospho-STAT1 and phospho-ERK1/2, the PVDF membranes were incubated with Restore PLUS Western Blot Stripping Buffer (#46430, Thermo Fisher Scientific) before probing for levels of total STAT1 and total ERK1/2.

### Statistical analyses

Two-tailed, unpaired Student t tests were used for statistical analyses, with the assumption of equal sample variance, with GraphPad Prism software. Differences with a p value of < 0.05 were considered statistically significant. ****p < 0.0001; ***p < 0.001, **p < 0.01; *p < 0.05. n.s., no significance.

## Supplementary Information

Supplementary Movie 1.

Supplementary Movie 2.

Supplementary Information 1.
