# Supplementary material for: SHP2 blockade enhances anti-tumor immunity via tumor cell intrinsic and extrinsic mechanisms
Source: Sci Rep. 2021 Jan 14;11:1399. doi: 10.1038/s41598-021-80999-x (PMC7809281; doi:10.1038/s41598-021-80999-x)

SHP2 blockade enhances anti-tumor immunity via tumor cell intrinsic and extrinsic mechanisms

Ye Wang[1], Morvarid Mohseni[1], Angelo Grauel[2], Javier Estrada Diez[1], Wei Guan[2], Simon Liang[1], Jiyoung Elizabeth Choi[2], Minying Pu[1], Dongshu Chen[1], Tyler Laszewski[2], Stephanie Schwartz[2], Jane Gu[1], Leandra Mansur[3], Tyler Burks[3], Lauren Brodeur[1], Roberto Velazquez[1], Steve Kovats[1], Bhavesh Pant[1], Giri Buruzula[2], Emily Deng[2], Julie T Chen[1], Farid Sari-Sarraf[4], Christina Dornelas[4], Malini Varadarajan[1], Haiyan Yu[1], Chen Liu[1], Joanne Lim[2], Huai-Xiang Hao[1], Xiaomo Jiang[2], Anthony Malamas[1], Matthew J LaMarche[5], Felipe Correa Geyer[1], Margaret McLaughlin[1], Carlotta Costa[6], Joel Wagner[1], David Ruddy[1], Pushpa Jayaraman[2], Nathaniel D Kirkpatrick[4], Pu Zhang[2], Oleg Iartchouk[3], Kimberly Aardalen[1], Viviana Cremasco[2], Glenn Dranoff[2], Jeffrey A Engelman[1], Serena Silver[1], Hongyun Wang[1], William D Hastings[2,7,*] , Silvia Goldoni[1,7,*]

[1] Oncology Disease Area, Novartis Institutes for BioMedical Research, Cambridge, United States;

[2] Exploratory Immuno-Oncology, Novartis Institutes for BioMedical Research, Cambridge, United States;

[3] Chemical Biology & Therapeutics, Novartis Institutes for BioMedical Research, Cambridge, United States;

[4] Analytical Sciences & Imaging, Novartis Institutes for BioMedical Research, Cambridge, United States;

[5] Global Discovery Chemistry, Novartis Institutes for BioMedical Research, Cambridge, United States;

[6] Oncology Disease Area, Novartis Institutes for BioMedical Research, Basel, Switzerland

[7] Co-corresponding authors: s.goldoni22@gmail.com, bill.hastings@novartis.com

*Contributed equally

Silvia Goldoni

William D Hastings

Novartis Institutes for BioMedical Research

250 Massachusetts Avenue, Cambridge, MA, 02139, United States

Legends of supplementary movies

Supplementary Movie 1

3D full view and section view of infiltrated PBMCs (Green) inside OVCAR-8 tumor spheroids (Red) after 24 hours of co-culture without any treatment via light sheet fluorescence microscopy imaging.

Supplementary Movie 2

3D full view and section view of infiltrated PBMCs (Green) inside OVCAR-8 tumor spheroids (Red) after 24 hours of co-culture with 20μM SHP099 treatment via light sheet fluorescence microscopy imaging.

Supplementary Figure 1

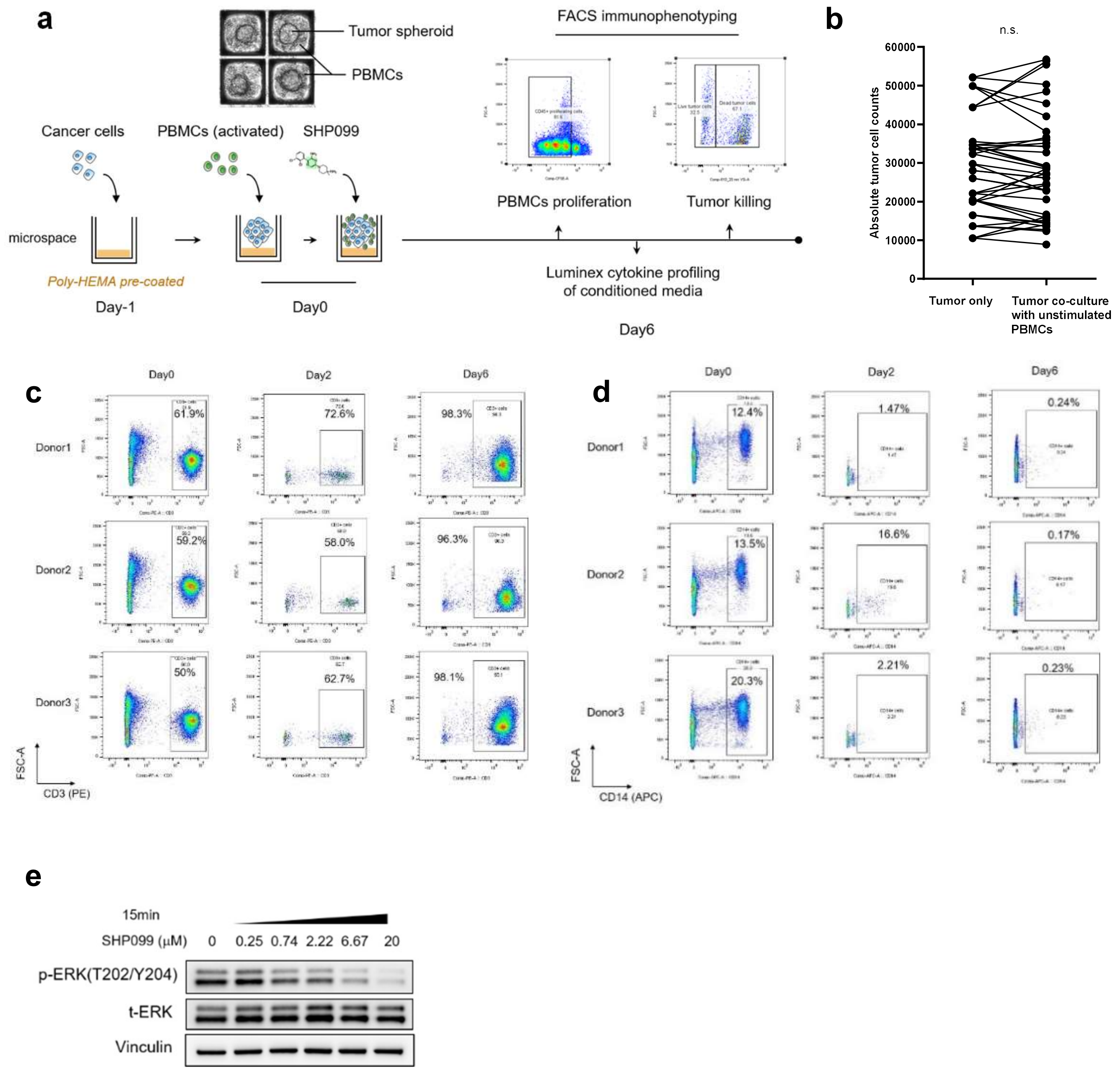

Supplementary Figure 1 (a) Flowchart showing the procedure of co-culturing tumor spheroids and PBMCs *in vitro*. **(b)** Absolute tumor cell counts of paired OVCAR-8 tumor only group and co-culture groups of OVCAR-8 spheroids with unstimulated PBMCs from multiple replicates with different donors. **(c)** Flow cytometry analysis of the percentage change of CD3-positive T cells over CD45-positive cells on Day 0, 2 and 6. CD3-positive cells are gated. Percentage of CD3-positive population is labeled. **(d)** Flow cytometry analysis of the percentage change of CD14-positive myeloid cells over CD45-positive cells on Day 0, 2 and 6. CD14-positive cells are gated. Percentage of CD14-positive population is labeled. **(e)** Immunoblotting of p-ERK1/2 in OVCAR-8 tumor spheroids after different treatments.

Supplementary Figure 2

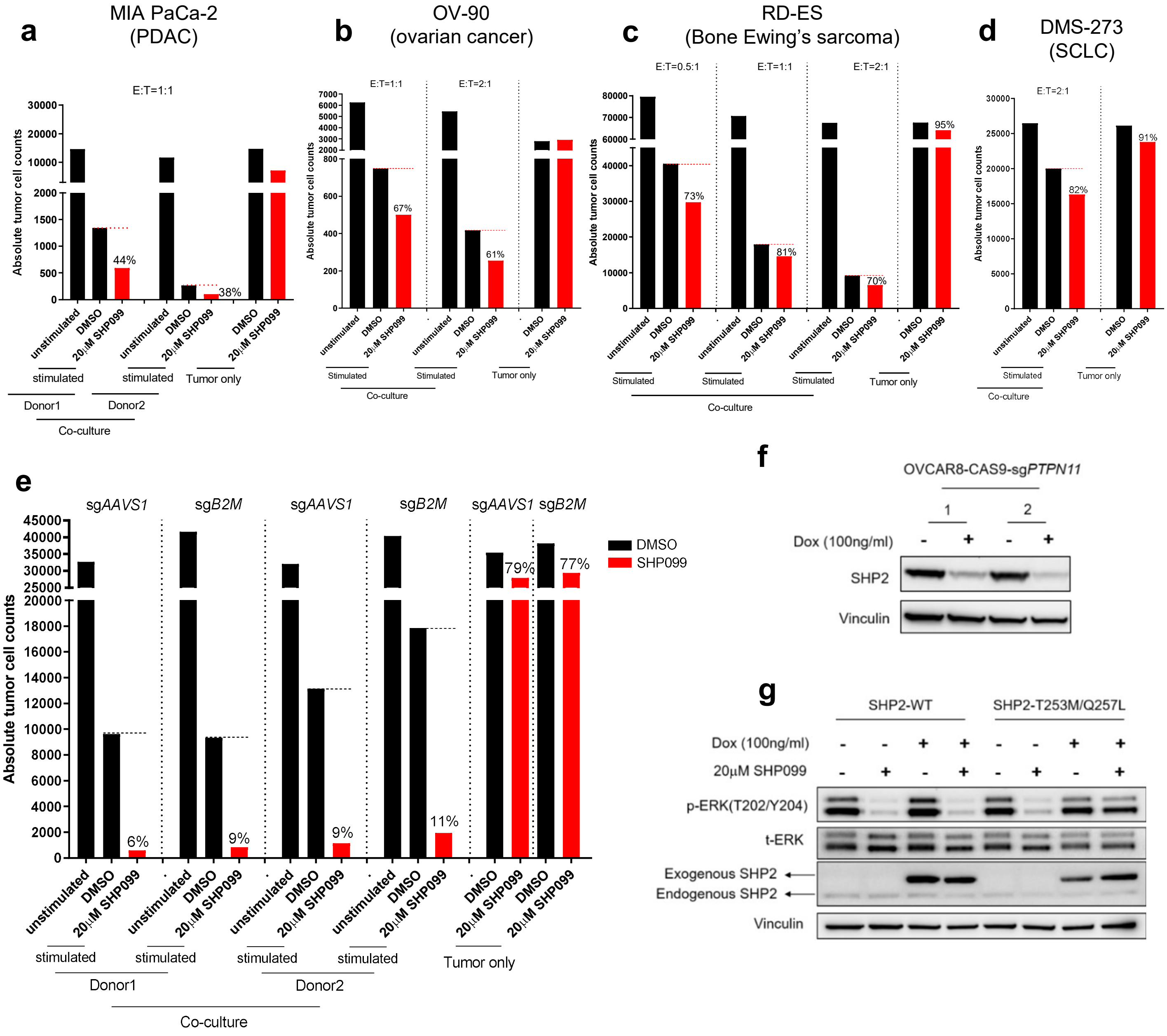

Supplementary Figure 2 (a) Absolute tumor cell counts from each well of 384-well Elplasia plate after 6 days co-culture of MIA PaCa-2 spheroids with human PBMCs (2 donors). Relative percentage of absolute tumor counts (SHP099 treated over DMSO group) is labeled. **(b)** Absolute tumor cell counts from each well of 384-well Elplasia plate after 6 days co-culture of OV-90 spheroids with human PBMCs (1 donor with 2 E:T ratio). Relative percentage of absolute tumor counts (SHP099 treated over DMSO group) is labeled. **(c)** Absolute tumor cell counts from each well of 384-well Elplasia plate after 6 days co-culture of RD-ES spheroids with human PBMCs (1 donor with 3 E:T ratio). Relative percentage of absolute tumor counts (SHP099 treated over DMSO group) is labeled. **(d)** Absolute tumor cell counts from each well of 384-well Elplasia plate after 6 days co-culture of DMS-273 spheroids with human PBMCs. Relative percentage of absolute tumor counts (SHP099 treated over DMSO group) is labeled. **(e)** Absolute tumor cell counts from each well of 384-well Elplasia plate after 6 days co-culture of OVCAR-8-sg*AAVS1* or OVCAR-8-sg*B2M* spheroids with PBMCs. Relative percentage of absolute tumor counts (SHP099 treated over DMSO group) is labeled. **(f)** Immunoblotting of SHP2 in OVCAR-8-CAS9-sg*PTPN11*-1&2 with and without 5 days of doxycycline treatment. **(g)** Immunoblotting of p-ERK1/2 and SHP2 in OVCAR-8-SHP2-WT and OVCAR-8-SHP2-T253M/Q257L with and without 5 days of doxycycline treatment.

Supplementary Figure 3

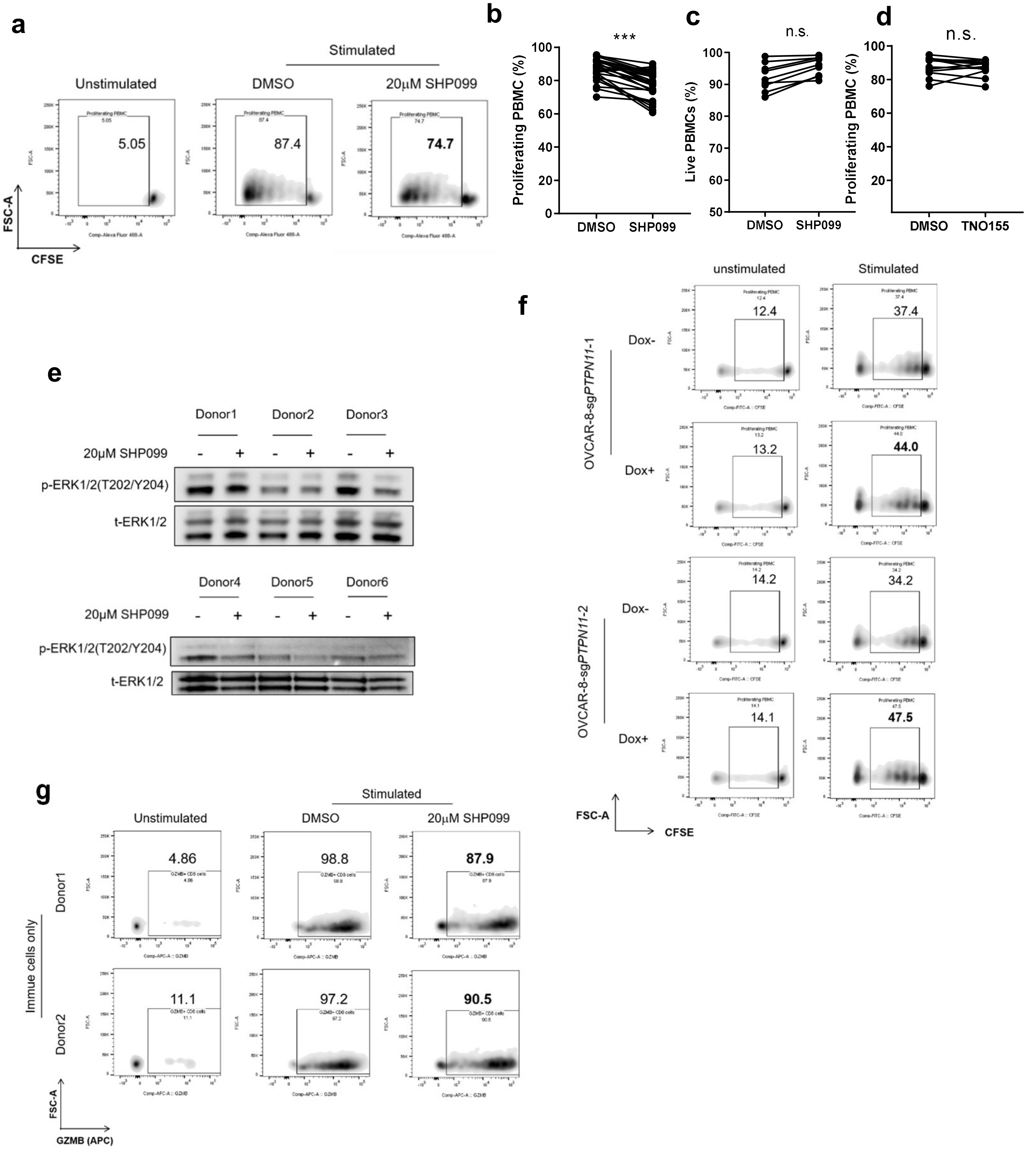

Supplementary Figure 3 (a) Flow cytometry analysis of T cell proliferation in PBMCs only culture with or without SHP099 treatment. T cells with diluted CFSE signal were gated. Percentage of T cell population with diluted CFSE signal was labeled. **(b)** Percentage of proliferating T cells from paired DMSO and SHP099 treated groups of PBMCs only from multiple replicates with different donors. **(c)** Percentage of live T cells from paired DMSO and SHP099 treated groups of PBMCs only from multiple replicates with different donors. **(d)** Percentage of proliferating T cells from paired DMSO and TNO155 treated groups of PBMCs only from multiple replicates with different donors. **(e)** Immunoblotting of p-ERK1/2 in T cells (from 6 donors) after 3 days activation by anti-CD3/CD28 beads and 15 min of SHP099 treatment. **(f)** Flow cytometry analysis of T cell proliferation in co-culture of OVCAR-8-CAS9-sg*PTPN11*-1 or OVCAR-8-CAS9-sg*PTPN11-2* spheroids with PBMCs. OVCAR-8 cells were treated with or without doxycycline (100ng/ml) for 5 days before co-culture. T cells with diluted CFSE signal were gated. Percentage of T cell population with diluted CFSE signal was labeled. **(g)** Flow cytometry analysis of intracellular staining of Granzyme B in PBMCs only culture with or without SHP099 treatment. Granzyme B-positive immune cells were gated. Percentage of Granzyme B-positive population was labeled.

Supplementary Figure 4

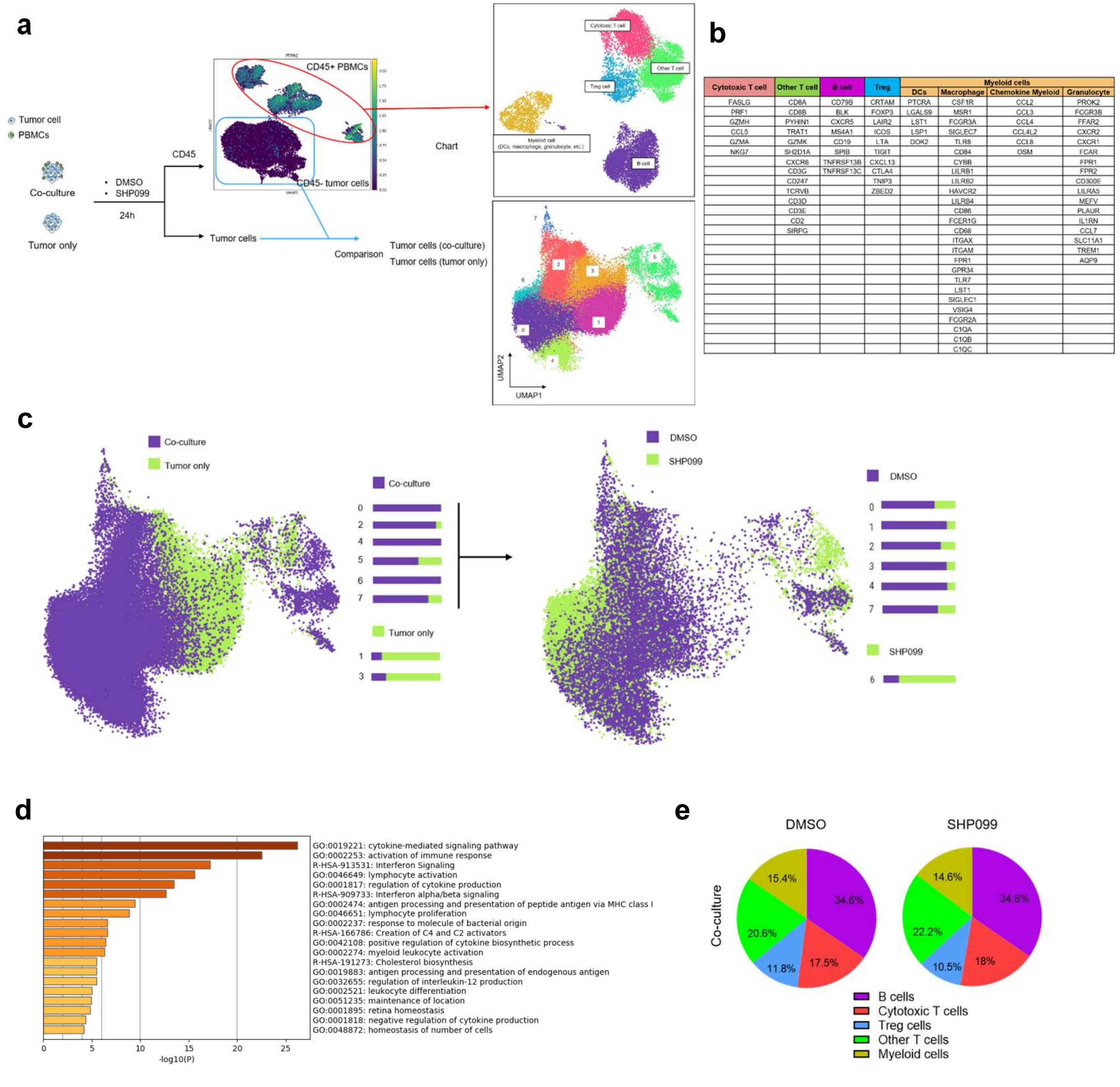

Cytotoxic T cell	Other T cell	B cell	Treg	Myeloid cells			
				DCs	Macrophage	Chemokine Myeloid	Granulocyte
FASLG	CD8A	CD79B	CRTAM	PTCRA	CSF1R	CCL2	PROK2
PRF1	CD8B	BLK	FOXP3	LGALS9	MSR1	CCL3	FCGR3B
GZMH	PYHIN1	CXCR5	LAIR2	LST1	FCGR3A	CCL4	FFAR2
CCL5	TRAT1	MS4A1	ICOS	LSP1	SIGLEC7	CCL4L2	CXCR2
GZMA	GZMK	CD19	LTA	DOK2	TLR8	CCL8	CXCR1
NKG7	SH2D1A	SPIB	TIGIT		CD84	OSM	FCAR
	CXCR6	TNFRSF13B	CXCL13		CYBB		FPR1
	CD3G	TNFRSF13C	CTLA4		LILRB1		FPR2
	CD247		TNIP3		LILRB2		CD300E
	TCRVB		ZBED2		HAVCR2		LILRA5
	CD3D				LILRB4		MEFV
	CD3E				CD86		PLAUR
	CD2				FCER1G		IL1RN
	SIRPG				CD68		CCL7
					ITGAX		SLC11A1
					ITGAM		TREM1
					FPR1		AQP9
					GPR34		
					TLR7		
					LST1		
					SIGLEC1		
					VSIG4		
					FCGR2A		
					C1QA		
					C1QB		
					C1QC		

Supplementary Figure 4 (a) Pipeline of scRNAseq analysis of tumor cells and immune cells from co-culture group and tumor cells from tumor only group. **(b)** Chart of gene expression sets for different immune cell populations. **(c)** Louvain clustering analysis of tumor cells from co-culture group and tumor only group (left), and from DMSO and SHP099-treated co-culture tumor cells (right). **(d)** Top 20 upregulated pathway signatures in tumor cells from cluster 6. **(e)** Percentage of different immune cell populations from DMSO and SHP099 treated co-culture groups.

Supplementary Figure 5

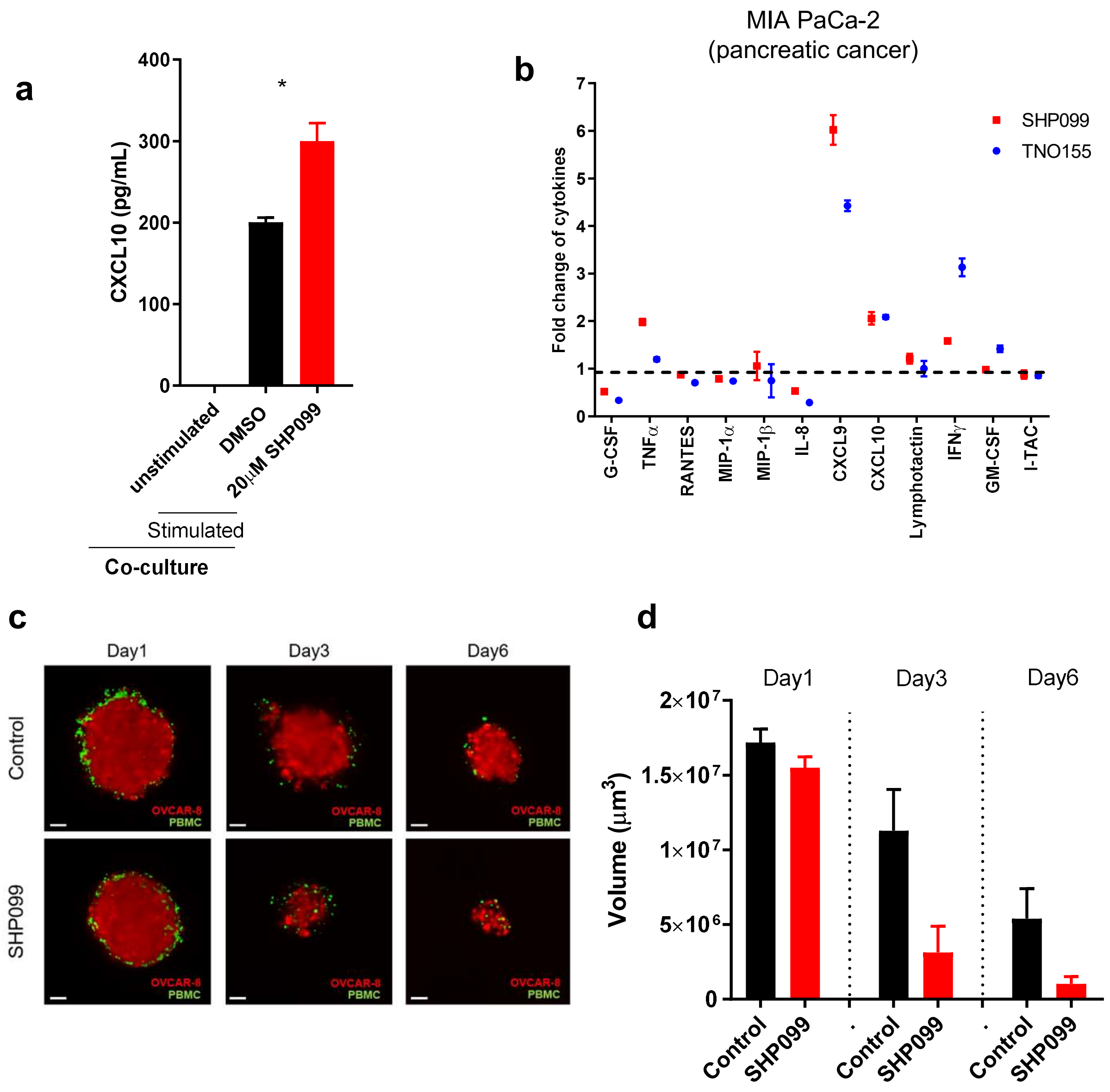

Supplementary Figure 5 (a) ELISA analysis of CXCL10 level in supernatant collected from 6 days co-culture of OVCAR-8 spheroids with PBMCs. **(b)** Luminex analysis of SHP099-induced fold change of a panel of cytokines (normalized to control group) in supernatants collected from co-culture of MIA PaCa-2 spheroids with PBMCs. **(c)** Light sheet microscopy imaging of infiltrated PBMCs (Green) inside OVCAR-8 tumor spheroids (Red) after 1 day, 3 days and 6 days of co-culture. Scale bar: 50 μm. **(d)** Histogram of mean tumor spheroids volume after 1 day, 3 days and 6 days of co-culture.

Supplementary Figure 6.

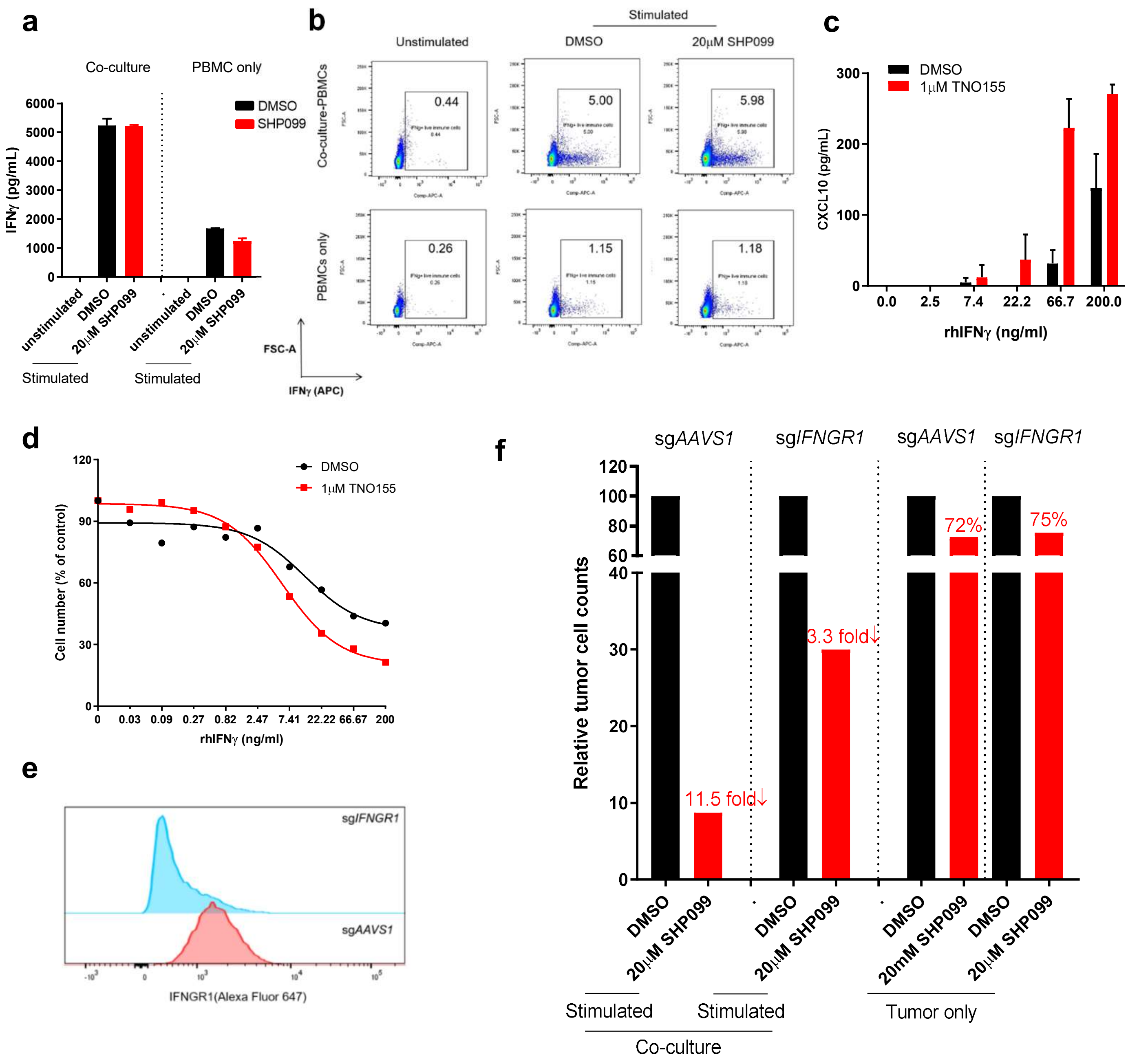

Supplementary Figure 6 (a) ELISA analysis of IFNγ level in supernatant collected from 2 days co-culture of OVCAR-8 spheroids with PBMCs or PBMCs only. **(b)** Flow cytometry analysis of intracellular staining of IFNγ in T cells from 2 days of co-culture and PBMCs only. IFNγ-positive cells are gated. Percentage of IFNγ-positive population is labeled. **(c)** ELISA analysis of CXCL10 level in supernatant collected from tumor spheroids treated with IFNγ of gradient concentration in the absence or presence of TNO155 for 6 days. **(d)** Dose response curve of OVCAR-8 tumor spheroids to IFNγ treatment in the absence or presence of TNO155 after 6 days. **(e)** Histogram of tumor cell surface IFNGR1 level of OVCAR-8-CAS9-sg*AAVS1* and OVCAR-8-CAS9-sg*IFNGR1*. **(f)** Absolute tumor cell counts from each well of 384-well Elplasia plate after 6 days co-culture of OVCAR-8-sg*AAVS1* or OVCAR-8-sg*IFNGR1* spheroids with PBMCs. Relative fold change of absolute tumor counts (SHP099 treated over DMSO group) is labeled.

Supplementary Figure 7.

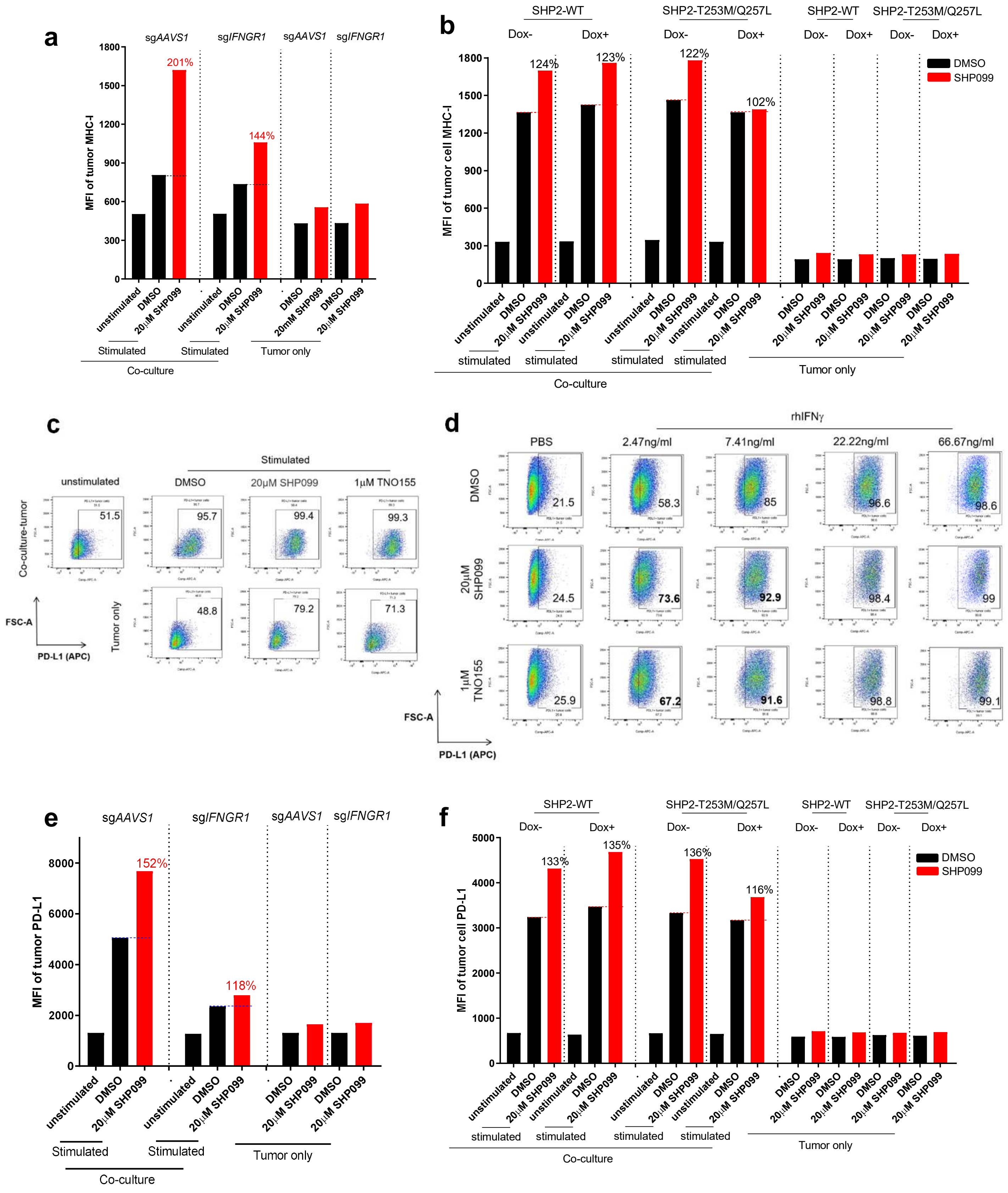

Supplementary Figure 7 (a) MFI of MHC class I in tumor cells after 6 days co-culture of OVCAR-8-sg*AAVS1* or OVCAR-8-sg*IFNGR1* spheroids with PBMCs. Relative percentage of MFI of tumor MHC I (SHP099 treated over DMSO group) was labeled. **(b)** MFI of MHC class I in tumor cells after 6 days co-culture of OVCAR-8-SHP2-WT or OVCAR-8-SHP2-T253M/Q257L spheroids with human PBMCs. OVCAR-8 cells were treated with or without doxycycline (100ng/ml) for 5 days before co-culture. Relative percentage of MFI of tumor MHC I (SHP099 treated over DMSO group) is labeled. **(c)** Flow cytometry analysis of cell surface staining of PD-L1 in tumor cells after 6 days of co-culture of OVCAR-8 spheroids with PBMCs or tumor only. PD-L1-positive cells were gated. Percentage of PD-L1-positive population was labeled. **(d)** Flow cytometry analysis of cell surface staining of PD-L1 in tumor spheroids treated with IFNγ of gradient concentration in the absence or presence of SHP099 and TNO155 for 6 days. PD-L1-positive cells were gated. Percentage of PD-L1-positive population was labeled. **(e)** MFI of PD-L1 in tumor cells after 6 days co-culture of OVCAR-8-sg*AAVS1* or OVCAR-8-sg*IFNGR1* spheroids with PBMCs. Relative percentage of MFI of tumor PD-L1 (SHP099 treated over DMSO group) was labeled. **(f)** MFI of PD-L1 in tumor cells after 6 days co-culture of OVCAR-8-SHP2-WT or OVCAR-8-SHP2-T253M/Q257L spheroids with human PBMCs. OVCAR-8 cells were treated with or without doxycycline (100ng/ml) for 5 days before co-culture. Relative percentage of MFI of tumor PD-L1 (SHP099 treated over DMSO group) is labeled.

Supplementary Figure 8.

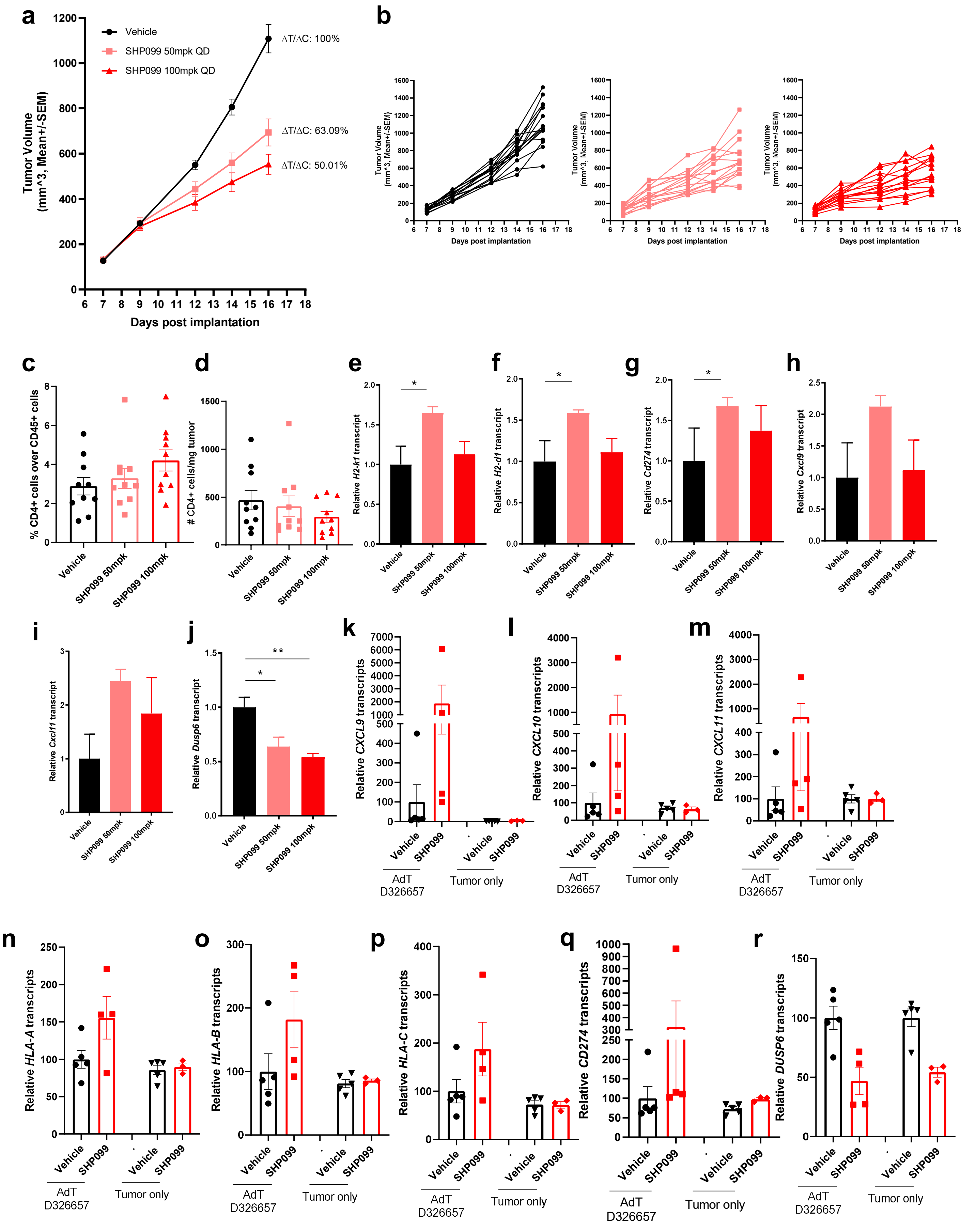

Supplementary Figure 8 **(a)** Tumor growth curve (expressed as tumor volume) of subcutaneous 4T1 tumors in NSG mice with different treatments. $\Delta T/\Delta C$ of last time point was labeled. **(b)** Spider plot of tumor growth curve (expressed as tumor volume) of subcutaneous 4T1 tumors in NSG mice with different treatments. **(c)** Flow cytometry analysis of the percentage of CD4+ T cells over CD45+ cells in tumor tissues with different treatments in 4T1 syngeneic model. **(d)** Flow cytometry analysis of the absolute number of CD4+ T cells per milligram tumor with different treatments in 4T1 syngeneic model. **(e-g)** Q-PCR analysis of the transcripts of mouse MHC Class I genes *H2-k1* **(e)**, *H2-d1* **(f)** and PD-L1 gene *Cd274* **(g)** in tumor cells enriched from 4T1 tumor tissues with different treatments. **(h-j)** Q-PCR analysis of the transcripts of *Cxcl9* **(h)**, *Cxcl11* **(i)** and *Dusp6* **(j)** in tumor cells enriched from syngeneic 4T1 tumor tissues with different treatments. **(k-r)** Q-PCR analysis of the transcripts of *CXCL9* **(k)**, *CXCL10* **(l)**, *CXCL11* **(m)**, *HLA-A* **(n)**, *HLA-B* **(o)**, *HLA-C* **(p)**, *CD274* **(q)** and *DUSP6* **(r)** in RD-ES tumor tissues from adoptive transfer model of human RD-ES with or without human PBMCs under different treatments.

Supplementary Figure 9.

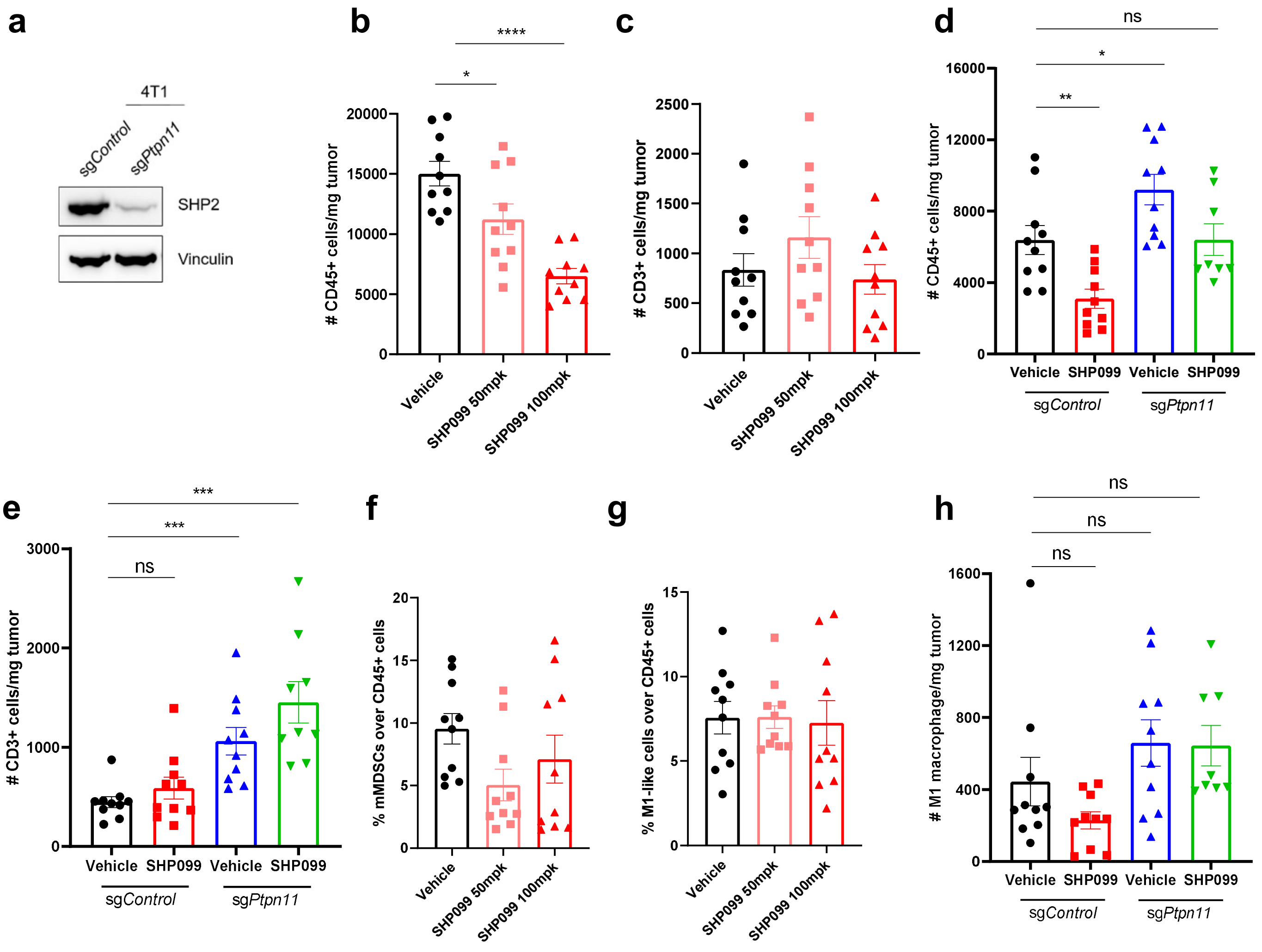

Supplementary Figure 9

(a) Immunoblotting of SHP2 in 4T1-CAS9-sg*Control* and 4T1-CAS9-sg*Ptpn11* cells. **(b)** Flow cytometry analysis of the absolute number of CD45+ immune cells per milligram tumor with different treatments in 4T1 syngeneic model. **(c)** Flow cytometry analysis of the absolute number of CD3+ T cells per milligram tumor with different treatments in 4T1 syngeneic model. **(d)** Flow cytometry analysis of the absolute number of CD45+ immune cells per milligram tumor in 4T1-sg*Control* and 4T1-sg*Ptpn11* syngeneic model with different treatments. **(e)** Flow cytometry analysis of the absolute number of CD3+ T cells per milligram tumor in 4T1-sg*Control* and 4T1-sg*Ptpn11* syngeneic model with different treatments. **(f)** Flow cytometry analysis of the percentage of mMDSCs over CD45+ immune cells with different treatments in 4T1 syngeneic model. **(g)** Flow cytometry analysis of the percentage of M1-like macrophage over CD45+ immune cells with different treatments in 4T1 syngeneic model. **(h)** Flow cytometry analysis of the absolute number of M1 macrophages per milligram tumor with different treatments in 4T1 syngeneic model.

Supplementary Figure 10.

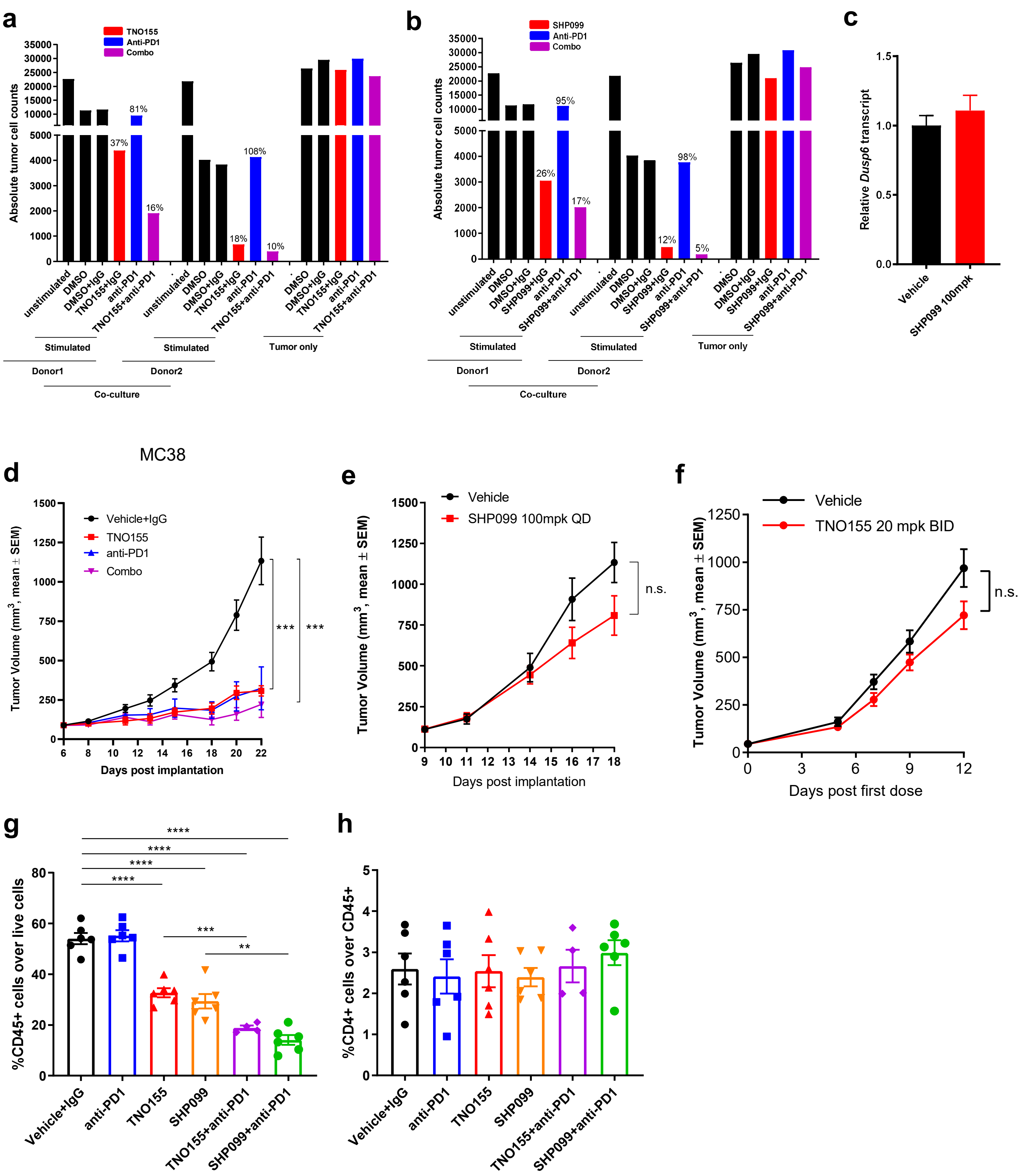

Supplementary Figure 10

(a, b) Absolute tumor cell counts from each well of 384-well Elplasia plate after 6 days co-culture of OVCAR-8 spheroids with human PBMCs (2 donors). **(a)** Relative percentage of absolute tumor counts (TNO155+IgG treated, anti-PD1 treated, TNO155+anti-PD1 treated over DMSO+IgG group) is labeled. **(b)** Relative percentage of absolute tumor counts (SHP099+IgG treated, anti-PD1 treated, SHP099+anti-PD1 treated over DMSO+IgG group) is labeled. **(c)** Q-PCR analysis of the transcripts of *Dusp6* in MC-38 tumor tissues from syngeneic mice. **(d)** Tumor growth curve (expressed as tumor volume) of subcutaneous MC38 tumors in MC38 syngeneic mice with different treatments (Vehicle+IgG, anti-PD1, TNO155 and TNO155+anti-PD1). Groups of Vehicle+IgG and anti-PD1 are the same as Figure 8C. **(e)** Tumor growth curve (expressed as tumor volume) of subcutaneous MC-38 tumors in NSG mice with different treatments (Vehicle and SHP099). **(f)** Tumor growth curve (expressed as tumor volume) of subcutaneous MC-38 tumors in NSG mice with different treatments (Vehicle and TNO155). **(g)** Flow cytometry analysis of the percentage of CD45+ immune cells over live cells in MC-38 syngeneic model. **(h)** Flow cytometry analysis of the percentage of CD4+ T cells over CD45+ immune cells in MC-38 syngeneic model.

Supplementary Figure 11.

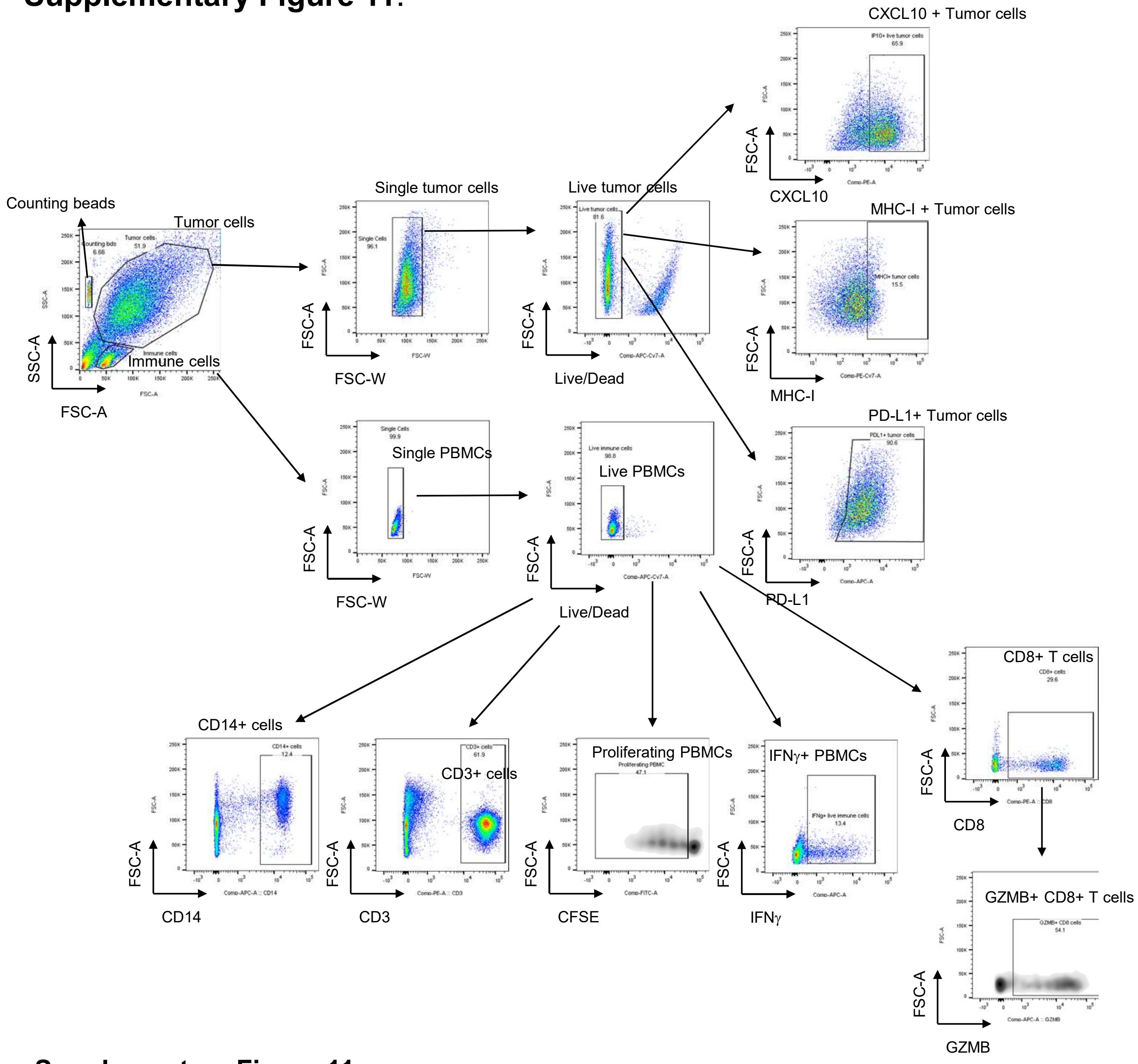

Supplementary Figure 11
Gating strategy for *in vitro* co-culture FACS analysis

Supplementary Figure 12.

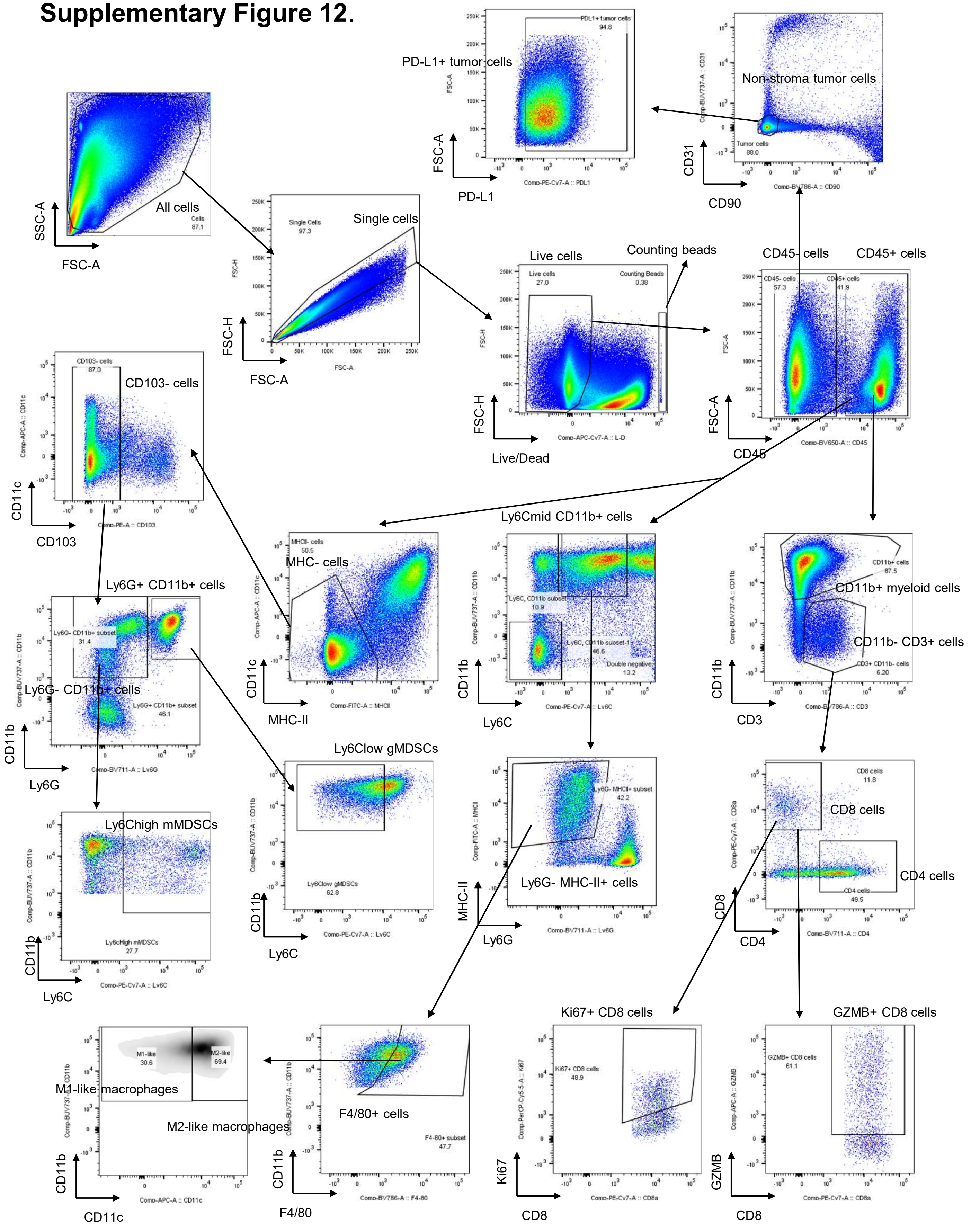

Supplementary Figure 12

Gating strategy for *in vivo syngeneic mouse models*

Original blots for Fig.4d

Fig.4d

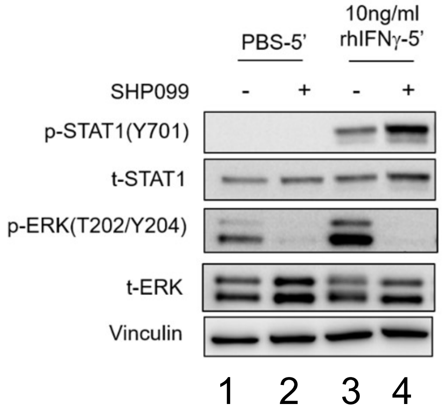

p-STAT1 (Y701)

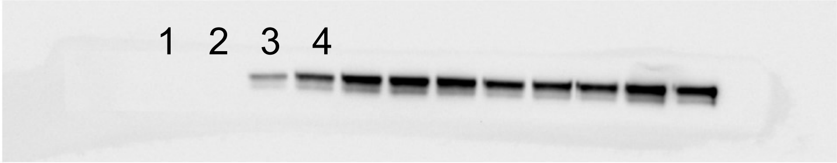

t-STAT1

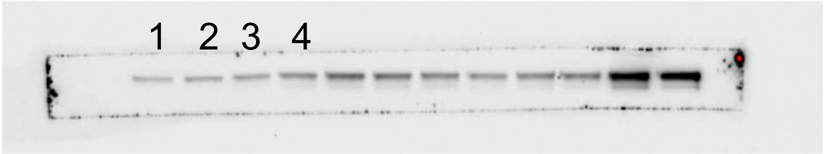

p-ERK (T202/Y204)

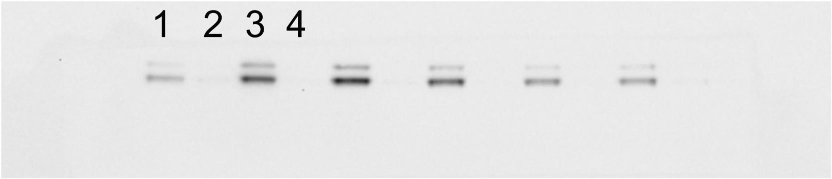

t-ERK

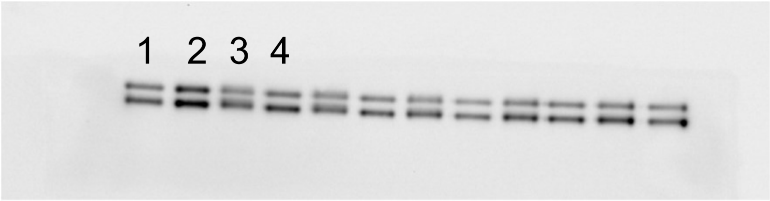

Vinculin

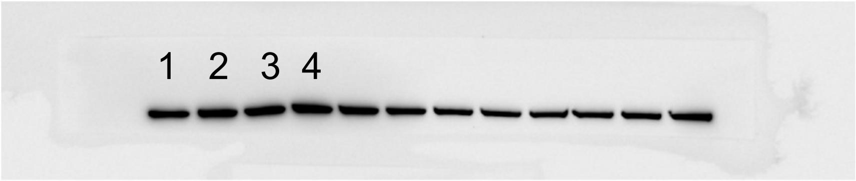

Original blots for Supplementary Fig.1e

Supplementary Fig.1e

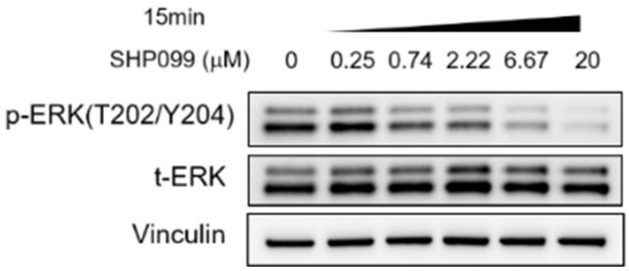

p-ERK (T202/Y204)

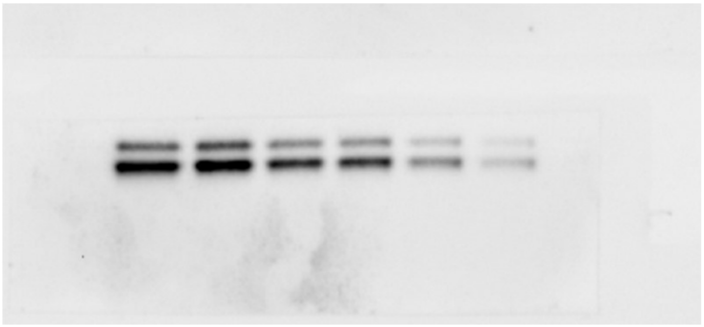

t-ERK

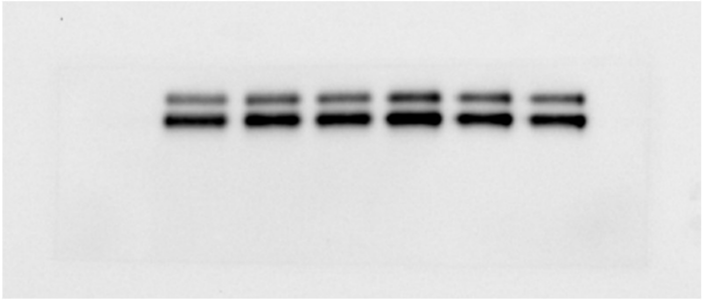

Vinculin

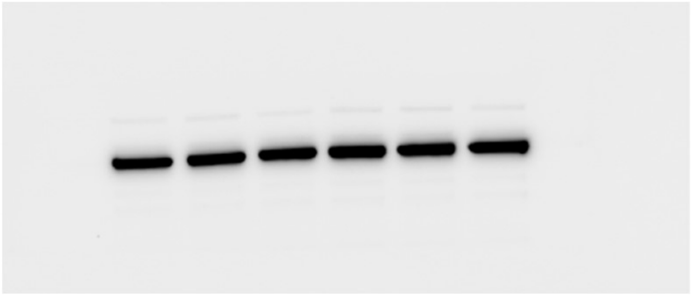

Original blots for Supplementary Fig.2f

Supplementary Fig.2f

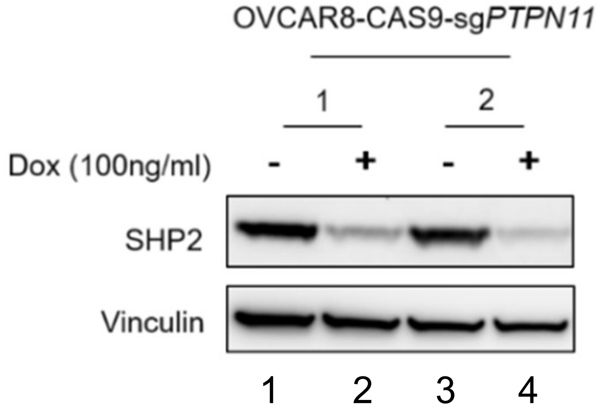

SHP2

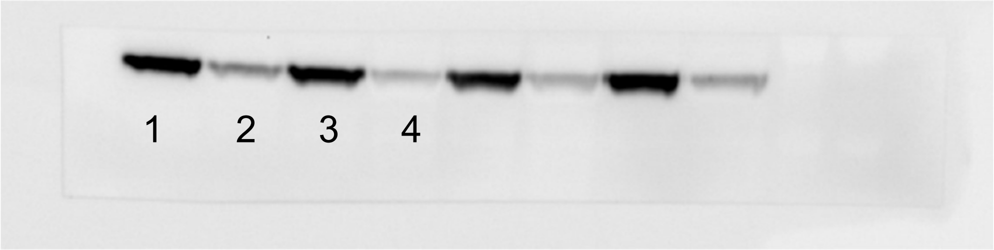

Vinculin

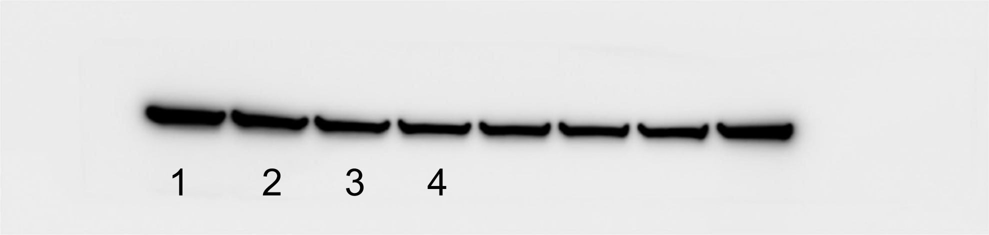

Original blots for Supplementary Fig.2g

Supplementary Fig.2g

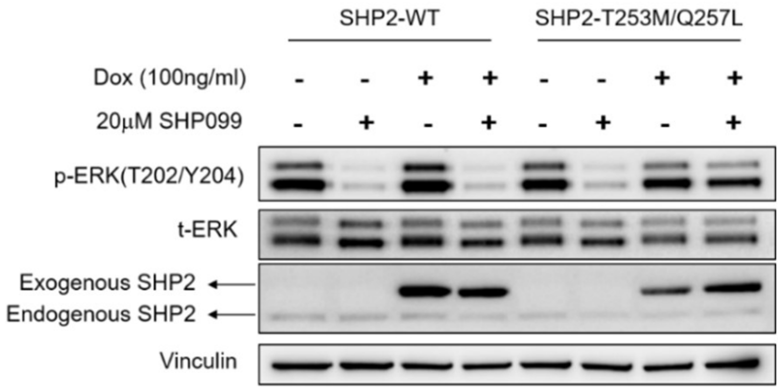

p-ERK (T202/Y204)

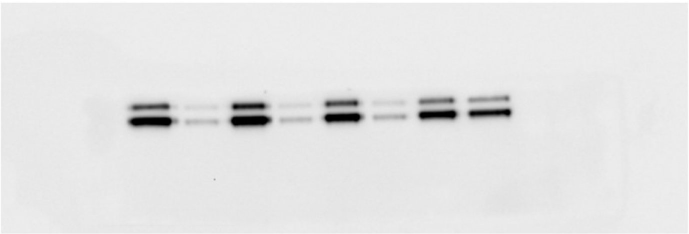

t-ERK

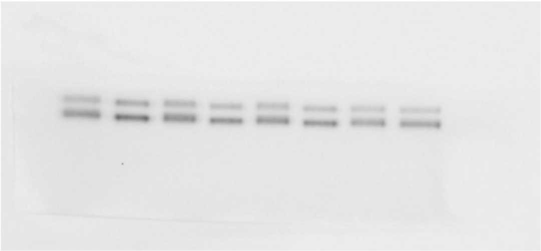

SHP2

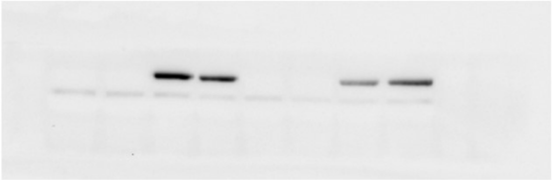

Vinculin

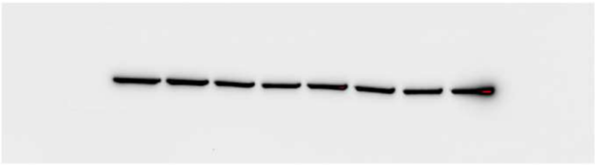

Original blots for Supplementary Fig.3e

Supplementary Fig.3e

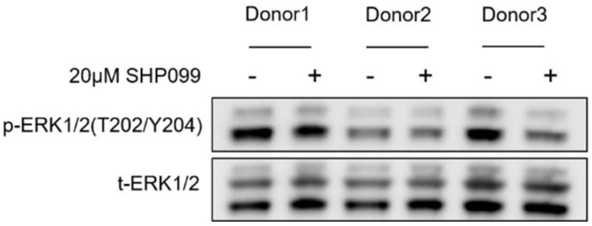

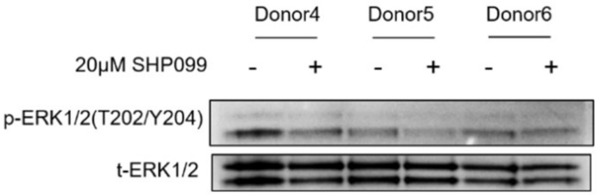

p-ERK (T202/Y204)

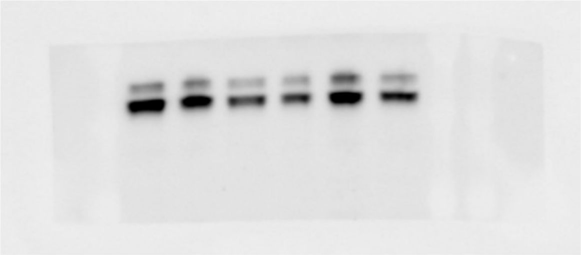

p-ERK (T202/Y204)

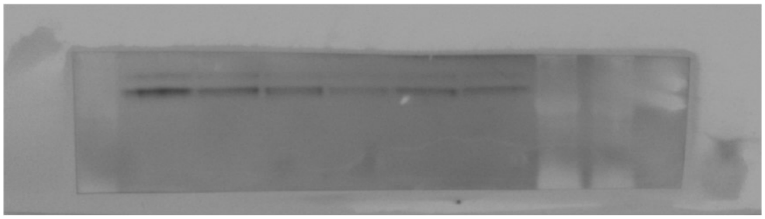

t-ERK

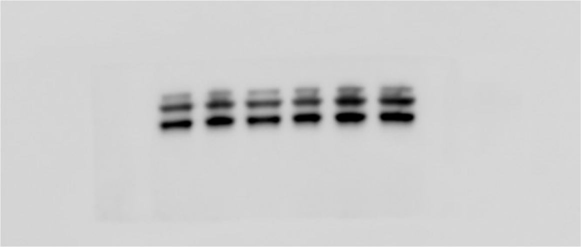

t-ERK

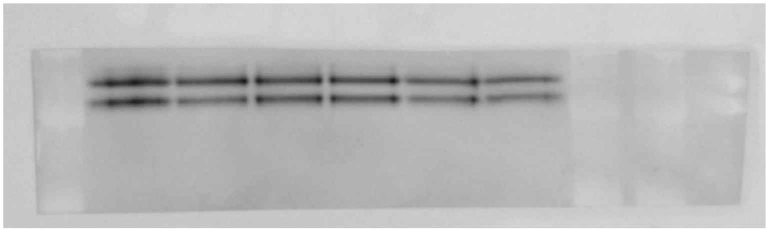

Original blots for Supplementary Fig.9a

Supplementary Fig.9a

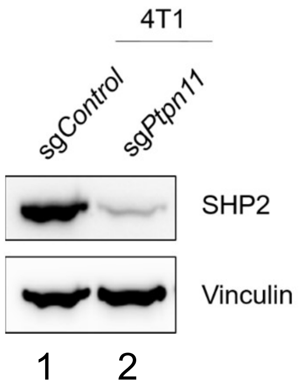

SHP2

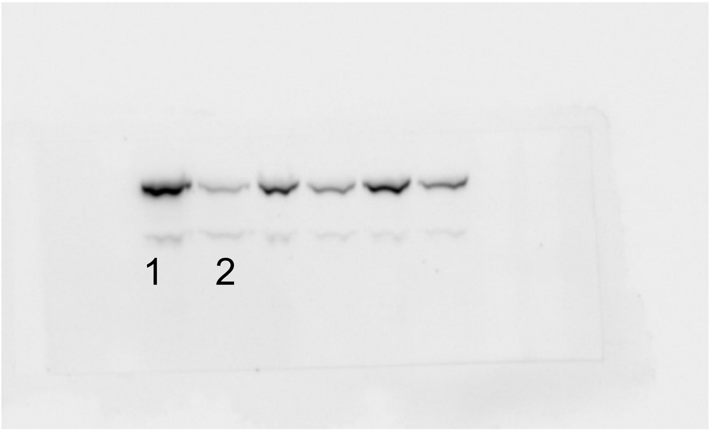

Vinculin

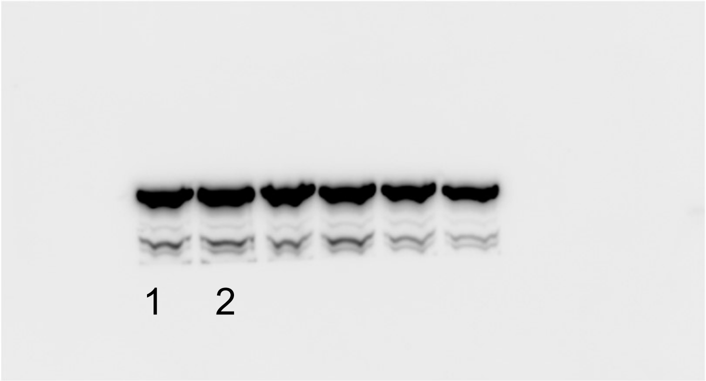

Supplement: Supplementary file 3 — Supplementary Information 1. [file 41598_2021_80999_MOESM3_ESM.pdf]
